# Attention networks and the intrinsic network structure of the human brain

**DOI:** 10.1002/hbm.25734

**Published:** 2021-12-09

**Authors:** Sebastian Markett, David Nothdurfter, Antonia Focsa, Martin Reuter, Philippe Jawinski

**Affiliations:** ^1^ Humboldt‐Universität zu Berlin Berlin Germany; ^2^ University of Bonn Bonn Germany

**Keywords:** attention network, attention network test, cognition, Connectome, functional connectivity

## Abstract

Attention network theory distinguishes three independent systems, each supported by its own distributed network: an alerting network to deploy attentional resources in anticipation, an orienting network to direct attention to a cued location, and a control network to select relevant information at the expense of concurrently available information. Ample behavioral and neuroimaging evidence supports the dissociation of the three attention domains. The strong assumption that each attentional system is realized through a separable network, however, raises the question how these networks relate to the intrinsic network structure of the brain. Our understanding of brain networks has advanced majorly in the past years due to the increasing focus on brain connectivity. The brain is intrinsically organized into several large‐scale networks whose modular structure persists across task states. Existing proposals on how the presumed attention networks relate to intrinsic networks rely mostly on anecdotal and partly contradictory arguments. We addressed this issue by mapping different attention networks at the level of cifti‐grayordinates. Resulting group maps were compared to the group‐level topology of 23 intrinsic networks, which we reconstructed from the same participants' resting state fMRI data. We found that all attention domains recruited multiple and partly overlapping intrinsic networks and converged in the dorsal fronto‐parietal and midcingulo‐insular network. While we observed a preference of each attentional domain for its own set of intrinsic networks, implicated networks did not match well to those proposed in the literature. Our results indicate a necessary refinement of the attention network theory.

## Graphical abstract

We assessed the relationship between attention networks and intrinsic connectivity networks. We show that different attention networks are all supported by the dorsal fronto‐parietal and the insular midcingulo‐network, casting doubts on most previous accounts on the network‐level representation of attention.

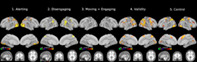

## 1 INTRODUCTION

In order to react adequately and to act purposefully in a dynamic and ever‐changing environment, the brain needs to prioritize information processing, for example, by anticipating when and where sensory information will appear, or by selecting more relevant over less relevant information. Attention refers to the cognitive function that guides the prioritization and selection of some at the expense of other information (Cowan, 1999; Posner & Fan, 2008). Converging evidence from single cell recordings, electrophysiology, and neuroimaging suggests that ongoing neural information processing is enhanced in a highly specific and targeted way when attention is shifted toward a certain location in the visual field (Brefczynski & DeYoe, 1999; Heinze et al., 1994; Kastner, Pinsk, De Weerd, Desimone, & Ungerleider, 1999; Luck, Chelazzi, Hillyard, & Desimone, 1997; Müller, Bartelt, Donner, Villringer, & Brandt, 2003) or toward task‐relevant stimulus features (Egner & Hirsch, 2005; O'Craven, Rosen, Kwong, Treisman, & Savoy, 1997; Rees, Frith, & Lavie, 1997). While the effects of attention become apparent in increased firing rates and BOLD activity in sensory areas, which process the currently attended information, the recruitment and control of attention signals is realized by neural systems that additionally include areas upstream on the cortical processing hierarchy (Posner & Dehaene, 1994; Posner & Petersen, 1990). Attention network theory assumes three largely independent systems that realize one out of three different types of attention: the alerting system initiates a state of increased arousal in direct anticipation of upcoming stimuli, the orienting system shifts the attentional focus to locations in space, and the control system selects and amplifies relevant information when distracting or task‐incompatible information is present (Posner & Petersen, 1990). The three systems are thought to dissociate neuroanatomically into independent “attention networks” (Posner & Rothbart, 2007). Evidence for the relative independence of the three attention systems comes from research with the attention network test (ANT; Fan, McCandliss, Sommer, Raz, & Posner, 2002). The ANT is a reaction time task that combines the flanker task (Eriksen & Eriksen, 1974) to study the attentional selection of relevant information at the expense of irrelevant distractors with the Posner cueing task (Posner, 1980) where briefly presented cues carry information when and where an upcoming target stimulus will appear. Behavioral indices of the efficiency of alerting, orienting, and control are uncorrelated (Fan et al., 2002) which is interpreted as an indication of independent systems. Moreover, genetic work points toward different genetic contributions and underlying susceptibility variants for the attention systems (Fan, Wu, Fossella, & Posner, 2001; Fossella et al., 2002; Reuter, Ott, Vaitl, & Hennig, 2007). Furthermore, neuroimaging work with the ANT has revealed nonoverlapping activation patterns for task contrasts that probe alerting, orienting, and attentional control (Fan, Mccandliss, Fossella, Flombaum, & Posner, 2005), adding further evidence for dissociable and presumably independent systems. More recent work, however, has documented partially overlapping activations for the different attention systems (Xuan et al., 2016), which is interpreted as the neural manifestation of interactions between the attention systems. Such interactions have also been observed at the behavioral level (Callejas, Lupiáñez, & Tudela, 2004; Fan et al., 2009). Finally, it has been hypothesized that the three attention systems dissociate at the level of intrinsic connectivity networks (Petersen & Posner, 2012). This, however, has not been addressed empirically.

Attention network theory uses the term “network” to refer to the distributed activation foci in the ANT. As the term “network” is also used to describe a set of intrinsic connectivity networks in the brain, it is imperative to clarify how attention networks and intrinsic connectivity networks relate to each other. Our understanding of brain networks has advanced majorly in the past years due to the increasing focus on brain connectivity. Several large‐scale networks that delineate along functional boundaries of the brain have been identified in spontaneous intrinsic BOLD fluctuations in the task‐free resting state (Fox & Raichle, 2007; Smith et al., 2013; van den Heuvel & Hulshoff Pol, 2010). Importantly, the intrinsic network architecture persists into task states and matches the topology of task‐evoked activations (Cole, Bassett, Power, Braver, & Petersen, 2014; Gordon, Stollstorff, & Vaidya, 2012; Nickerson, 2018; Smith et al., 2009). If the three attention systems were actually independent networks, we would assume that each system activates a distinct or distinct group of intrinsic connectivity network (ICN). This has also been suggested previously, for instance, that the three attention networks segregate within an “extended fronto‐parietal network” (Xuan et al., 2016), that the orienting network corresponds to a dorsal and a ventral fronto‐parietal network and the attention control network to a distinct fronto‐parietal and an insular‐opercular network (Petersen & Posner, 2012). The fronto‐parietal and insular‐opercular network have also been discussed regarding their role in alerting (Sadaghiani & D'Esposito, 2014). Some of these previous propositions, however, rely only on anecdotal arguments and appear in conflict with each other.

At present, it is unclear how the idea of three separable and independent attention networks as activated by the ANT is reflected in the overall network structure of the brain. We designed the current study to directly probe the spatial correspondence between ICN and the three attention networks. Since attention networks are often equated with the activation patterns elicited by the ANT at the measurement level, such comparison would also clarify the relationship between two distinct concepts for which the term “network” is widely used.

We first recorded resting‐state fMRI data in order to delineate ICN and second recorded task fMRI data from the same participants with the most recent version of the ANT (the revised ANT, (Fan et al., 2009; Xuan et al., 2016). We made use of recent developments by the Human Connectome Project to achieve high spatial precision through multimodal surface matching (Robinson et al., 2018) and minimal spatial smoothing (Glasser et al., 2016). We expect to replicate previous findings with the ANT: We expect behavioral indices for the efficiency of different attention systems to be uncorrelated and we expect significant activations at previously reported voxel locations.

We probed the relationship between ICN and different attention contrasts through separate spatial regression analyses (Gordon et al., 2012). We first ask whether attention systems dissociate at the ICN level. Since the regressions' beta weights quantify bivariate spatial correspondence, a dissociation of the attention systems at the ICN level would be reflected in a nonsignificant or significantly negative correlation of the beta weights from different attention contrasts. Furthermore, we would expect that no single ICN contributes to all attention contrasts. We ask second, if certain ICN contribute specifically to any of the attention systems, in order to obtain evidence in favor of any of the previous proposals how the attention networks relate to different ICN.

## 2 METHODS

### 2.1 Participants

We recruited *N* = 86 healthy young adults (age: M = 26.17 years, *SD* = 5.41 years; *n* = 39 females, *n* = 47 males) through flyer advertisements on campus, mailing lists, and announcement in undergraduate psychology classes. Participants were screened during a telephone interview to meet the following inclusion criteria: Native‐level proficiency in German, right‐handedness, and age between 18 and 35 years. We targeted an equal amount of male and female participants. Participants were excluded when they indicated past or present psychiatric or neurological illness, psychotropic substance use in the past 6 months, or any contraindication to MRI. Participants reported to have normal or corrected‐to‐normal vision during the experiment. Informed written consent was obtained prior to enrollment in the study. Participants were remunerated with the usual rate of 10 EUR/hr (i.e., 25 EUR for the entire study) or its equivalence in course credit, if desired by the participant. The study protocol was in accordance with the Declaration of Helsinki and approved by the ethics committee of the University Hospital Bonn.

### 2.2 Attentional network test

**FIGURE 1:**
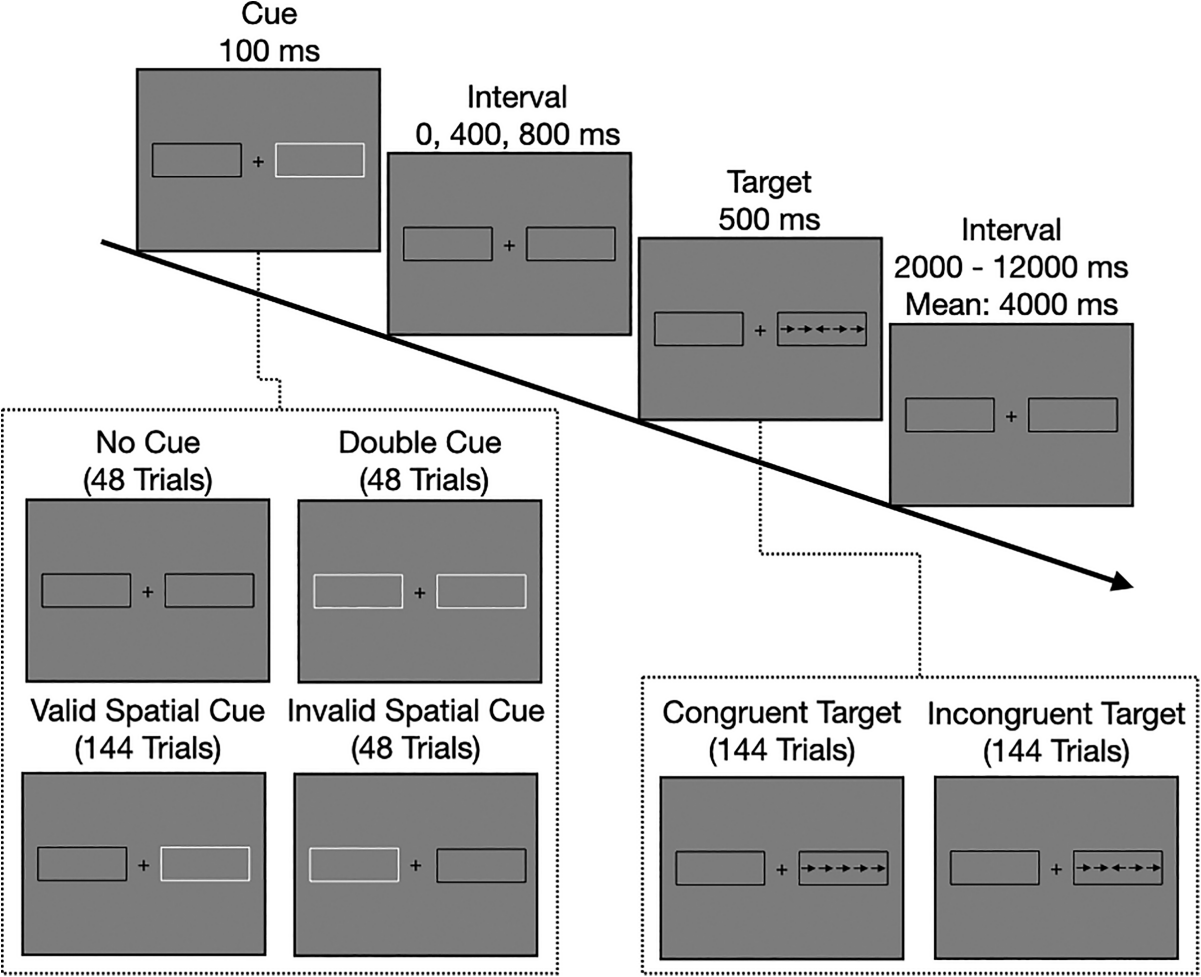
Schematic overview over stimuli and stimulus timing in a typical trial sequence. Each trial started with a 100 ms presentation of either no cue, a double cue, or a spatial cue. After a cue‐target interval of 0, 400, or 800 ms, five arrows were flashed for 500 ms as target stimulus. Participants indicated via button press whether the central arrow pointed to the left or to the right. Flanking arrows were either congruent or incongruent (half of the trials each). Target offset and onset of the next cue were spaced by a jittered interval (mean interval across trials: 4,000 ms, range: 2,000–12,000 ms). Targets appeared either at the cued position (valid spatial cues) or at uncued position (invalid spatial cue). A total of 288 trails was presented in four runs

We adapted the Attentional Network Test in its revised form (Xuan et al., 2016). The ANT‐R combines a spatial cueing with a flanker task and is the standard protocol to activate different attentional systems in the brain. We administered a total of 288 trials in four runs of 72 trials each. A typical trial sequence is shown in Figure 1. The ANT‐R follows a 4 × 2 design with the factors cueing condition (no cue, double cue, valid spatial cue, and invalid spatial cue) and target (congruent flanker, incongruent flanker). Activation maps and behavioral indices for the attention networks were computed by contrasting different cue and target conditions as described below (see task analysis and behavioral analysis). Each run lasted for 420 s, leading to a total time of around 30 min for the whole experiment.

Throughout each run a fixation cross was presented in the middle of the screen, surrounded by a rectangle on its left and right side (the rectangles subtended 4.69° of visual angle to both sides). The fixation cross and the rectangles remained visible during the whole run. In every trial, arrows were presented in one of the rectangles: An arrow in the center (target) was surrounded by two arrows each on the left and the right side (flankers). Each arrow subtended 0.58° of visual angle and the distance between arrows was 0.06° of visual angle. The arrows pointed either to the left or to the right and the five arrows could either be congruent (i.e., the target arrow pointed to the same direction as the surrounding flankers) or incongruent (i.e., the target arrow pointed to the opposite direction as the flankers). Participants were instructed to select as fast and accurately as possible the direction of the middle arrow by either pressing a button in the left or the right hand. In some trials, a cue was presented before the flankers appeared via brightening of one or both of the rectangles. As a spatial cue, only one of the rectangles flashed, while a brightening of both rectangles (double cue) served as a temporal cue. Spatial cues could either be valid, that is, the arrows were presented in the rectangle that brightened, or invalid, that is, the arrows were presented in the opposite rectangle. A short interval was implemented between cue and flanker presentation.

Each trial consisted of three phases: a cue phase (100 ms), a short interval (0, 400 or 800 ms, equally distributed), and a target phase (500 ms). The different conditions were spread across two blocks consisting of 144 trials: Of the 144 trials, one sixth, that is, 24, were no cue, double cue, and invalid spatial cue trials, respectively. The other half, that is, 72 trials, consisted of valid spatial cues. Each cue type was followed by each other cue type equally often (Fan et al., 2009). The 24 combinations of interval between cue and target phase, flanker type (congruent or incongruent) and target location (left or right rectangle) were randomized for each cue condition. The interval between offset of target and onset of the next trial was distributed systematically between 2,000 and 12,000 ms with a mean of around 4,000 ms (for details see Fan et al., 2009). While the target was only presented for 500 ms, participants had additional 1,200 ms to press the button after offset of the target, leading to a total time frame of 1,700 ms to respond.

The experiment was programed with Presentation software version 20.1 (Neurobehavioral Systems, Inc., Albany, CA) and presented via a projector in the MR scanner. The projection screen had a resolution of 1,024 × 768px (24 × 18 cm) and the distance between screen and participants' eyes was around 62 cm. Participants took part in a short training block consisting of 10 trials outside of the MR scanner to get familiar with the setup.

### 2.3 Image acquisition

All MR images were acquired in a single session on a Siemens 3T Prisma equipped with a 32 channel head coil at the Berlin Center for Advanced Neuroimaging between March and December 2019. We adopted MR sequences from the HCP‐Lifespan project (Harms et al., 2018). The following protocols were acquired in a fixed order: (a) T1‐weighted structural (Multiecho MPRAGE, voxel size 0.8 mm isotropic, time to repeat TR = 2.4 s, time to echo TE = 22 ms, flip angle 8°), (b) T2‐weighted structural (SPACE, voxel size 0.8 mm isotropic, TR = 3.2 s, TE = 563 ms, flip angle 120°), (c) BOLD rfMRI (multiband echoplanar, 72 slices, 805 volumes, TR = 800 ms, voxel size 2 mm isotropic, TE = 37 ms, flip angle 52°, A‐P encoding direction) including two spin echo fieldmaps (A‐P and P‐A encoding), and (d) tfMRI in four runs with run‐specific spin echo fieldmaps and the same pulse sequence as for rfMRI 4) Diffusion‐weighted images (DWI). DWI data will not be part of the present report. A reference image without multiband acceleration was acquired for each functional run.

### 2.4 Preprocessing

We adapted the HCP minimal preprocessing pipelines (github.com/Washington‐University/HCPpipelines) for structural and functional preprocessing (Glasser et al., 2013). If not stated otherwise, we used version 4.1 of the pipelines, Freesurfer 6.0.0, and FSL 6.0.1 under Linux Debian 10. Structural images (T1 and T2) were corrected for gradient distortions, aligned, brain extracted, bias field corrected, and registered to MNI space using nonlinear transformation. Structural images where then further processed with HCP's Freesurfer pipeline with improved brain extraction, alignment, and adjustment of the white matter surface. The Freesurfer output was converted to Nifti and Gifti files and used to create a brain mask for all further analyses. Cortical surfaces were then registered to template space based on cortical folding (MSMsulc, Robinson et al., 2018) and downsampled to the 32k_LR surface space. All functional data (rfMRI and task fMRI) and the corresponding field maps were processed with the fMRIVolume pipeline, which included correction for gradient distortions, motion, EPI image distortions, co‐registration with the T1 structural image, and normalization to MNI volumetric space. All transformations were applied in one step. Functional data were then intensity normalized to their global 4D mean and masked. The resulting volume timeseries were further processed with the fMRISurface pipeline to create individual CIFTI dense timeseries grayordinate files by resampling subcortical gray matter voxels to standard subcortical parcels and by partial‐volume‐weighted and cortical‐ribbon‐constrained‐mapping of cortical gray matter voxels onto standard surface vertices. In this step, we applied light volume‐ and surface‐based smoothing with a Gaussian filter with 2 mm full width at half maximum.

Resting state timeseries were processed further to remove artifacts. Each participants' volumetric rfMRI timeseries were first run through FSL's Multivariate Exploratory Linear Optimized Decomposition into Independent Components (MELODIC) tool (ve3.15) and then processed using FSL Fix (v1.06.15). We used a classifier that had been trained on the HCP young adult sample as distributed with FIX. Automatic component classification worked excellent despite small differences in acquisition parameters between our data and the training data. Manual inspection indicated that no component had to be re‐labeled. Artifactual components were regressed out together with the six head motion parameters and their first temporal derivatives. The cleaned rfMRI time series were then converted to grayordinates as described above. The FIX pipeline was run on a 12‐core Mac Pro (High Sierra 10.12.6) machine using R (v3.3.3), Matlab (v2018b), and HCPpipelines (v4.2.1). Relevant R‐packages were used in the respective version mentioned in the FIX documentation and re‐compiled when needed.

### 2.5 Independent component analysis

We identified ICN at the group level by running a group ICA in MELODIC after concatenating all participants' FIX‐cleaned dense timeseries grayordinate files in time and reducing the data matrix into a 1,609 dimensional subspace. We requested 27 components, after estimating the ICA's dimensionality in Matlab using HCP code (icaDim.m). Four artifactual noise components were identified through visual inspection and the remaining 23 components were kept for further analyses.

**FIGURE 2:**
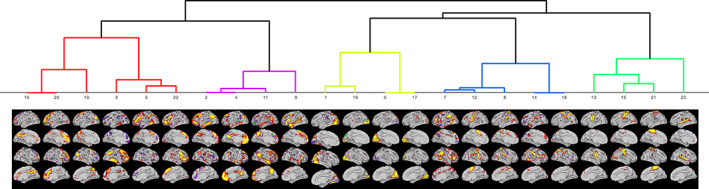
Thresholded statistical maps of independent components and their grouping into ICN through hierarchical clustering of associated time courses. The components in the red cluster belong to the executive control and fronto‐parietal network, the components in magenta to the default mode and language networks, the components in light green represent the (visual) occipital network, the components in blue the midcingulo‐insular and dorsal fronto‐parietal “attention” networks, and the components in the darker green cluster represent the somatomotor and auditory networks. The numbers of the components correspond to the order of the ICA output (ordered by variance explained), the ordinal position in the figure was determined by the clustering

We labeled these ICN based on (a) the similarity of associated time courses obtained through hierarchical clustering of the IC time courses based on the time courses' full correlation network matrices using the Ward method in FSLnets (v0.6.3) (see Figure 2), (b) pairwise comparison of each thresholded and binarized (|z| > 3) ICN with published atlases (the Cole‐Anticevic cortical and subcortical partition, the Yeo 7 and 17 cortical networks, and the Power partition, see Table T1), and (c) visual inspection and comparison with a detailed map of cortical areas (see Figure S1).

### 2.6 Task analyses

First level analyses were performed in SPM12 (www.fil.ion.ucl.ac.uk/) using a general linear model. Surface images were converted to “fake‐volumetric” nifti‐images using wb_command. Condition specific regressors were created by convolving a train of delta functions with SPM's canonical hemodynamic response function. Following the ANT's four (cues) by two (targets) design, separate regressors reflected the onsets of the following events: Congruent targets following double cues, congruent targets following valid cues, congruent targets following invalid cues, congruent targets following no cues, incongruent targets following double cues, incongruent targets following valid cues, incongruent targets following invalid cues, incongruent targets following no cues. In addition to these eight regressors, we added one additional regressor with the onsets of error trials, 12 regressors with the 6 head motion parameters and their temporal derivatives, and one constant per run. The final design matrix contained (8 + 13 + 1) * 4 columns.

Linear weighted contrasts were computed on the estimated beta images to derive the attention network maps (see Fan et al., 2005; Xuan et al., 2016): Alerting network: Double cue minus no cue (across target conditions). Control network: all incongruent targets minus all congruent targets (across cue conditions). The orienting network was operationalized via the Validity effect (invalid cue minus valid cue, across target conditions), which is a combination of disengaging attention from an invalid location (invalid cue minus double cue, the Disengaging effect) and moving and engaging the attentional focus to a validly cued location (valid cue minus double cue, the Moving + Engaging effect). Individual contrast images were back‐converted to cifti‐files and then passed on to second level group analysis.

We used the Sandwich Estimator (SwE) Toolbox for SPM12 (Guillaume, Hua, Thompson, Waldorp, & Nichols, 2014) for group‐level analyses of individual contrast images to assess activation of attentional systems across all participants. SwE's main application is longitudinal and repeated measures neuroimaging data, but SwE is also suitable for more simple designs like ours. We used the modified SwE procedure with a small sample size correction (type c) and a wild bootstrapping procedure with 999 bootstraps. The family‐wise error was corrected at the cluster level (*p* <.05) with a cluster‐forming threshold of *p* <.001. The thresholding of the activation maps was also done for display purposes: All follow‐up analyses on spatial correspondence with ICN made use of the unthresholded maps.

In order to establish that our adaptation of the ANT led to similar activations as reported in previous work, we repeated the second level analysis in SPM12 with volumetric data (4 mm smoothing kernel). We created volumetric masks for each of the five attention contrast with 8 mm spheres around the peak voxel locations reported in Xuan et al. (2016) and controlled the familywise error at the voxel level within these masks. The masks are shown in the supplementary Figure S3 and Table T2.

### 2.7 Spatial regression

Our main question focuses on the spatial relationship between intrinsic ICN and the different attentional “networks” as activated by the ANT‐R. We used a multiple spatial regression approach (Gordon et al., 2012) to predict group‐level ANT‐activation maps from the 23 group‐level ICN. Separate models were estimated for each activation map. Unthresholded activation maps (*z*‐images) were reshaped to column vectors that included all cortical vertices and subcortical voxels. These vectors served as criterion in the regression analyses. On the predictor level, all 23 nonartifactual unthresholded IC maps were reshaped into a grayordinate * component matrix. Ordinary least square regressions were fitted using Matlab's fitlm function. Possible confounds due to collinearity were ruled out by inspecting condition indices and variance decomposition proportions from the predictor matrix. Effect sizes for individual ICN were calculated as partial regression coefficients by residualizing each ICN from all remaining ICN, fitting a linear regression model, and obtaining the adjusted *R^2^*.

### 2.8 Behavioral analysis

We analyzed reaction times and error rates to compute behavioral indices of the different attention networks. For reaction time analyses, all error trials and responses outside a response window of 1,700 ms after target onset were excluded (see Xuan et al., 2016). Behavioral indices were computed as differences between experimental conditions: Alerting: no cue minus double cue, Orienting: Invalid Cue minus Double Cue (Disengaging), Double Cue minus Valid Cue (Moving + Engaging), and Invalid Cue minus Valid Cue (Validity Effect), and Control: Incongruent minus congruent target. We calculated five (attention contrasts) * two (reaction times, error rates) one‐sample *t*‐tests to test whether the behavioral index differed significantly from zero. To test for independence of different attention systems, we computed linear correlations between all five indices. We also quantified behavioral interactions between alerting and control, orienting and control, and the validity effect and control as detailed in Fan et al. (2009). Significance was assessed by separate two‐way ANOVAs (no cue vs. double cue by congruent vs. incongruent for the alerting‐control interaction, double vs. valid cue by congruent vs. incongruent for the orienting‐control interaction, and valid vs. invalid cue by congruent vs. incongruent for the validity‐control interaction).

### 2.9 Final sample

We had to exclude two participants because of incidental findings. The final rfMRI sample included *N* = 84 (mean age M = 26.34, *SD* = 5.35, *n* = 38 female, *n* = 46 male). Six participants were excluded from the task analysis for committing an excessive number of errors in the ANT (*n* = 4), large artifacts in the task fMRI data (*n* = 1), and incomplete task fMRI data (*n* = 1). The final tfMRI sample included *N* = 78 subjects (mean age M = 26.19, *SD* = 5.34, *n* = 35 female, *n* = 43 male).

## 3 RESULTS

### 3.1 Behavioral results

Mean differences and test statistics for reaction times and committed errors are presented in Table 1. As expected, the presence of temporal and valid spatial cues led to faster reaction times while invalid cues and incongruent flankers led to slower responses. The same pattern was also visible in the error rates, except for the alerting contrast where the difference was not statistically significant.

**TABLE 1:** Behavioral main effects and interactions for reaction times and error rates

Attention effects										
	Reaction times					Accuracy				
	M	*SD*	T	*df*	*p*	M	*SD*	T	*df*	*p*
A	33.18	31.29	9.36	77	<.001	0.11	2.87	0.33	77	.74
D	33.37	27.61	10.67	77	<.001	0.9	3.07	2.60	77	.01
M	39.66	25.31	13.84	77	<.001	0.43	2.51	1.53	77	.13
V	73.02	35.65	18.09	77	<.001	1.34	3.01	3.92	77	<.001
C	150.07	51.44	25.77	77	<.001	3.83	5.01	6.75	77	<.001

Interactions										
	M	*SD*	*F*	*df*	*p*	M	*SD*	*F*	*df*	*p*
A*C	−10.82	48.08	3.95	1,77	.05	−0.96	5.52	2.35	1,77	.13
O*C	15.77	39.02	12.74	1,77	<.001	1.09	4.54	4.46	1,77	.04
V*C	13.47	48.67	5.98	1,77	.02	1.29	5.70	3.98	1,77	.05

*Note*: A, alerting; D, disengaging; M, moving and engaging; V, validity effect; C, control; A*C, alerting by control; O*C, orienting by control; V*C, validity by control.

As expected, behavioral indices for the major attention contrasts (alerting, validity, control) where not correlated (see Table 2). Significant correlations were only obtained for contrasts that shared a reference condition.

**TABLE 2:** Correlations between behavioral indices of attention network efficiency

	Alerting	Disengaging	Moving	Validity	Control
Alerting		.296**	−.469**	−.103	−.049
Disengaging	.271*		. 094	. 707**	.024
Moving	−.545**	−.431**		.636**	−.025
Validity	−.177	.660**	.394**		.001
Control	.063	−.024	.263	.195	

*Note*: The upper triangle gives correlation coefficients for behavioral indices based on reaction times. The lower triangle gives corresponding correlations based on accuracy.

* *p <*.05.

** *p <*.01.

For reaction times, a 4 (cue conditions) by 2 (congruency) ANOVA revealed significant main effects for cue (*F*_(3,231)_ = 188.92, *p* <.001), congruency (*F*_(1,77)_ = 647.89, *p* <.001), and a significant interaction (*F*_(3,231)_ = 3.52, *p* = .016). For error rates, a similar ANOVA model revealed significant main effects for cue (*F*_(3,231)_ = 5.55, *p* = .001), congruency (*F*_(1,77)_ = 45.54, *p* <.001), but no significant interaction (*F*_(3,231)_ = 1.87, *p* = .135). The significant interaction suggests a modulation of the congruency effect by cue condition. Running 2 * 2 post hoc ANOVAs, we confirmed interaction effects between orienting and control and between validity and control for reaction times (see Table 1).

In sum, we replicated previous findings on the behavioral independence and interactions of attention networks (Fan et al., 2009; Xuan et al., 2016).

### 3.2 Intrinsic connectivity networks

Figure 2 shows the 23 group‐level ICN and the hierarchical clustering of their time courses. High resolution maps of the 23 ICN are published on BALSA. Pairwise comparison of each thresholded and binarized ICN with published atlases and a list of implicated cortical regions are documented in Table T1 and Figure S1. The springgreen cluster on the very right of Figure 2 represents the larger *somatomotor network (SMN)*: ICN #13 (“mouth” network) as well as #15 and #21 (“hand” network). ICN #23 corresponds to the auditory network. Components #7, #12, and #8 in the blue cluster on the left overlap with networks labeled cingulo‐opercular, ventral attention, and salience network in different published partitions. These labels refer often to the same *midcingulo‐insular network* (Uddin, Yeo, & Spreng, 2019). The other two components in the blue cluster (#14 and #18) include most prominently areas along the intraparietal sulcus and correspond to the *dorsal fronto‐parietal* “attention” *network*. The four components in the yellowgreen cluster in the middle represent the larger *visual network*: IC #6 is the primary or peripheral, and #1, #17, and #19 are the secondary or central visual network. Components #2, #4, and #11 in the magenta cluster on the left represent the *default mode network*. The other component in the magenta cluster (ICN #9) is left‐lateralized and overlaps with the *language network* first described in Ji et al. (2019). The Power et al. (2011) parcellation interprets this component as the ventral attention network, which has been criticized and corrected by Ji et al. (2019). Implicated regions are more consistent with a role in language than attention: auditory cortex, premotor area 55b, and includes inferior prefrontal cortex with areas 44 and 45 that represent Broca's area. Components #3, #5, and #22 in the red cluster on the very left correspond to the *lateral fronto‐parietal network*. The remaining three components #10, #16, and #20 are more difficult to label. The hierarchical clustering grouped the networks together on a hierarchy level, which allowed a clear interpretation for all other components. Pairwise spatial comparisons with published templates indicate overlap with different task positive networks. Inspecting the precise boundaries of the components indicate involvement of higher order association areas including inferior and dorsolateral prefrontal cortex. We will therefore use the label *executive control network* when referring to these three components.

### 3.3 Attentional networks

**FIGURE 3:**
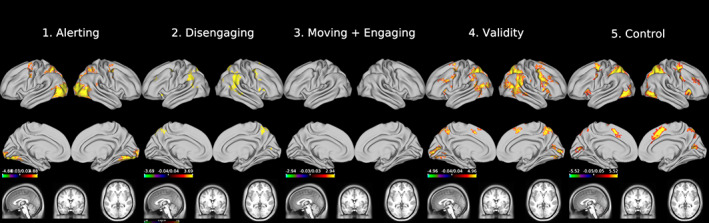
Group level activation maps (surface data) of the five contrasts. All maps show Gaussianized *t*‐statistics, thresholded at *p* <.05, FWE‐corrected. Underlying anatomical images are HCP's midthickness surface mesh and HCP's average T1‐weighted structural image. We did not find significant clusters in the volumetrtic subcortical data, possibly due to the small smoothing kernel of 2 mm

Figure 3 shows activation maps for all task contrasts (i.e., the attention networks) Alerting (a), disengaging (b), moving and engaging (c), the validity effect (d), and control (e). At large, activation patterns are consistent with the patterns reported in Xuan et al. (2016): We found significant activation increases at 11/37 reported peak coordinates for alerting (set‐level inference *c* = 15, *p* <.001), 8/20 for the validity effect (set‐level inference *c* = 11, *p* <.001), and 15/25 for control (set‐level inference *c* = 16, *p* <.001). We found only significant activation increase at one location for disengaging and none for moving and engaging. The replicated peak locations implicate all activation clusters reported by Xuan et al. (2016), except for the left anterior insula and the locus coeruleus for alerting, and the left inferior occipital gyrus for the validity effect. We refer to the Supporting Information for volumetric versions of the activation maps, depictions of the masks, and detailed statistics.

### 3.4 Spatial regression

We fitted five linear models that regressed the spatial distribution of task‐evoked activity in the five attention contrasts onto the 23 ICN. The 23 predictor variables did not show any signs of collinearity (all condition indices <1.61, all variance decomposition proportions <.5, see Figure S2). The adjusted coefficients of determination (*R* ^2^) of the regression models indicated that the spatial brain‐wide topology of the 23 ICN accounted for 53.52% of the variance in alerting (RMSE = 1.03), 53.6% of the variance in disengaging (RMSE = 1.005), 29.84% of the variance in moving and engaging (RMSE = .9261), 61.19% of the variance in the validity effect (RMSE = 1.0774), and 65.96% of the variance in control (RMSE = 1.195).

If the attention systems dissociated at the level of ICN, we would expect the beta coefficients from the spatial regression analyses to be uncorrelated (or negatively correlated). The correlations are presented in Table 3. The significant positive correlations between alerting, validity, and control indicate that ICN that contribute stronger to one of the attention systems contribute also to the other attention systems, placing doubts on the presumed dissociation of attention systems at the intrinsic connectivity level.

**TABLE 3:** Correlations between beta estimates from the spatial regression for five attention contrasts

	Alerting	Disengaging	Moving	Validity	Control
Alerting		.286	−.869*	.677*	.645*
Disengaging			.037	.874*	.659*
Moving				−.451*	−.381
Validity					.773*
Control					

* *p <*.001.

**FIGURE 4:**
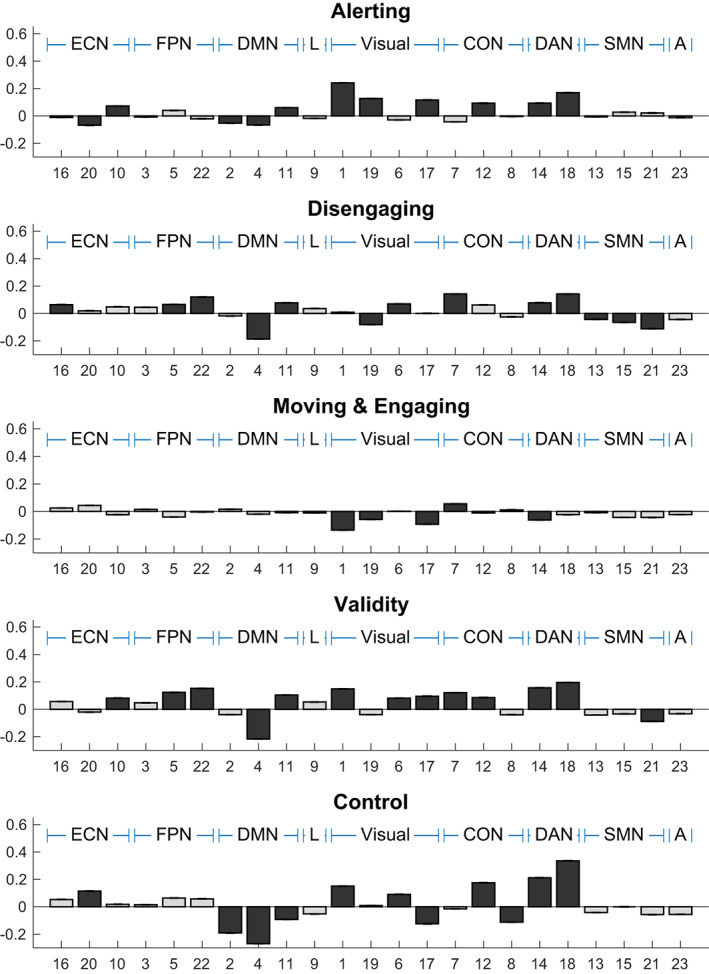
Results from the spatial regression analyses. Different panels correspond to different attention contrasts. Bars represent regression beta weights for the 23 ICN. The numbers on the *x*‐axis reflect the order in the MELODIC. Bars in darker gray indicate components that explained at least 1% of variance in the criterion

Figure 4 shows regression weights for each ICN and task contrast. We will discuss involvements of ICN that explain at least 1% to the overall variance in the attention contrasts.

We first asked how many different ICN were involved in each attention contrast. Eleven ICN explained at least 1% of the variance in alerting, thirteen in disengaging, five in moving and engaging, thirteen in validity, and eleven in control. We then assessed whether implicated ICN were particularly enriched in any of the higher‐level groups identified in the hierarchical clustering: Permutation testing revealed that the visual (*p* = .0394), dorsal attention (*p* <.001), and default mode network (*p* <.001) were recruited by alerting, the somatomotor (*p* <.001), and dorsal attention network (*p* <.001), by disengaging, and the visual (*p* <.001) and dorsal attention network (*p* = .042) by moving and engaging. The dorsal attention network (*p* <.001) was engaged by the validity effect. The visual (*p* = .0376) and dorsal attention network (*p* <.001) were engaged while the default mode network (*p* <.001) was suppressed by control.

We next asked whether any ICN was involved in all attention functions (alerting, control, and at least one of the orienting contrasts). We found that ICN #4 and #11 (parts of the default mode network). ICN #1 and #17 (parts of the visual network), ICN #12 (midcingulo‐insular network), and ICN #14 and #18 (the dorsal attention network) were recruited by all attention functions. From the two default mode network components, ICN #4 (default mode network) was consistently suppressed during all attention functions while ICN #11 was engaged by alerting and the orienting contrasts, but suppressed during control.

We then examined whether any ICN was specifically involved in only one attention contrast. This was the case for eight ICN: ICN #16 (executive control network) was only activated by disengaging, and ICN #5 and #22 (fronto parietal network) and ICN #7 (midcingulo‐insular network) were only activated by orienting (disengaging and the validity effect). ICN #8 (midcingulo‐insular network) was suppressed by control, ICN #13 and #15 (somatomotor network) were suppressed by disengaging, and ICN #21 (somatomotor network) was suppressed by the validity effect. We also examined which ICN did not contribute to any attention contrasts: This was the case for ICN #4 (fronto‐parietal network), ICN #9 (language network), and ICN #23 (auditory network).

Finally, we examined which ICN showed the largest positive contribution to each attention contrast.

ICN with largest positive individual contributions were ICN #1 (primary visual network) for alerting (21.13%), ICN #7 (midcingulo‐insular network) for disengaging (9.81%) and moving and engaging (2.66%), and IC #18 (dorsal attention network) for the validity effect (6.84%) and for control (14.39%).

### 3.5 Overlap between attentional networks

**FIGURE 5:**
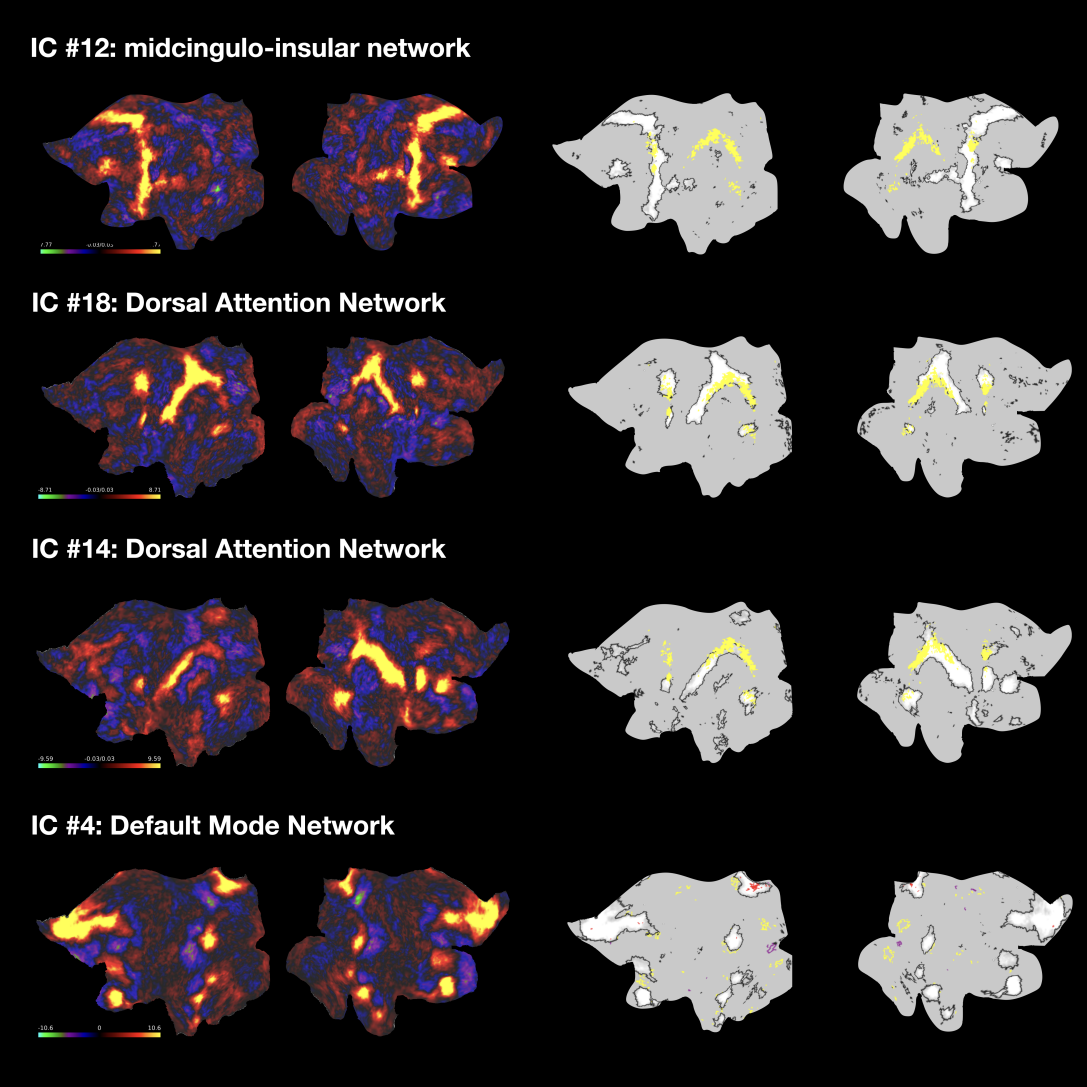
Overlapping activation in the midcingulo‐insular, dorsal fronto‐parietal “attention,” and default mode network. The flat maps on the left show the unthresholded IC maps. The flat maps on the right show overlapping activations for alerting, validity, and control (yellow, top three panels) and overlapping deactivations for validity and control (red, bottom panel). The outline of significant deactivations for validity (purple) and control (yellow) is given for comparison. The thresholded IC maps are shown in white‐gray with black outlines for spatial reference

The spatial regression analysis pointed at seven ICN components that were recruited by all attention domains. Leaving out the two visual components that are likely to be the target of top down modulation and one component in the default mode network that showed opposing contributions (positive and negative) to different attention networks, we are left with four components which are detailed in Figure 5. The right column shows grayordinate‐level overlaps of significant activations for alerting, validity, and control: All three attention systems activated overlapping areas in superior parietal cortices and the dorsal stream along the intraparietal sulcus, which are key areas of the dorsal fronto‐parietal network.

Within superior premotor cortex, all three attention systems overlapped in the frontal eye field (dorsal fronto‐parietal network), and the premotor eye field as well as area 6a (both midcingulo‐insular network). The default mode network (IC #4) was suppressed in all attention contrasts. The bottom panel of Figure 5 shows grayordinate level overlap of significant deactivations for the validity and the control contrast (there was no deactivation for alerting above the statistical threshold). While the control contrast led to more widespread deactivations that traced the outline of default mode component #4, the deactivations for the validity contrast were more focal, yet mostly at the same grayordinate locations as control deactivations.

### 3.6 Alternative parcellation

We used our own ICA‐based parcellation for the assessment of spatial overlap between ANT networks and ICN. For an easier comparison with other parcellations, we computed bivariate spatial overlap between all five ANT contrasts and the Yeo networks (7 networks and 17 networks, Yeo et al., 2011). Analyzing the Yeo networks yielded highly similar results: Across ANT networks, the correlation coefficients quantifying spatial correspondence with ICN were positively correlated (particularly for alerting, the validity effect, and control). For both partitions, the dorsal attention network showed highest and most consistent contributions to alerting, validity, and control. Of note, the ventral attention network of the Yeo‐7 partition and the salience‐ventral‐attention network of the Yeo‐17 partition did not contribute substantially to any ANT contrast. Detailed results are documented in Figures S9 and S10, Table T2.

## 4 DISCUSSION

Attention network theory distinguishes three types of attention that work together in any given situation but are realized by separate networks. The present work utilized the widely used ANT and successfully replicated previous work on the topology of activations and behavioral independence of the attention systems. It has been proposed that the attention networks dissociate in the intrinsic connectivity architecture of the brain, and different propositions have been made how these attention networks correspond to the various ICN described in the literature, yet none of them has been addressed comprehensively. To achieve a better understanding of how attention is represented at the network level, we reconstructed 23 independent components from high‐resolution resting‐state data and utilized a spatial regression approach (Gordon et al., 2012) to study the topological correspondence between ICNs and the activation of the attention networks. We did not find evidence for a dissociation at the intrinsic network level: If an ICN increased its activation during one type of attention, it was also more likely to activate during other types of attention. We also did not find a clear correspondence between attention networks and single ICNs. Instead, we observed that each attention system activates components in multiple ICNs, that the majority (around) 87% of all components contribute to at least one attention system, and that the components activated by different attention networks overlap substantially. The dorsal fronto‐parietal network, which included large stretches of superior parietal as well as premotor and inferior parietal cortices and areas along the dorsal visual stream (IC #14 and IC #18) was recruited by all attention systems. Further, parts of the midcingulo‐insular network which included premotor and midcingular cortex, the paracentral lobule, and the posterior as well as the insular and frontal opercula (IC #12) were also recruited by all attention systems, while parts of the default mode network which included the typical midline regions (IC #4) were deactivated by all attention contrasts. This is suggestive of a shared neural resource among the three different attention systems in the form of at least three different ICN.

We grouped the 23 components into nine larger ICN and assessed whether the different attentional networks preferably recruited components from any of these ICN. We observed that the dorsal fronto‐parietal network as a whole was recruited by all attention systems. Regarding other ICN, we observed some differences: The somatomotor network as a whole was suppressed during disengaging from an invalidly cued location, the default mode network was suppressed during the attentional control of irrelevant information but also involved in establishing alertness. The visual network was engaged by alerting and control. As these findings are based on a direct comparison between the intrinsic network structure and activated attention networks, they provide important insights into the correspondence of ICN and the attention networks, which may clarify previous propositions, which we are going to discuss in the following.

### 4.1 Attentional networks: the extended fronto‐parietal network hypothesis

It has been suggested that the fronto‐parietal network underlies attention (Toro et al., 2008), which is supported by correlations between network properties of the fronto‐parietal network and behavioral indices of attention (Markett et al., 2014; Visintin et al., 2015). Accordingly, Xuan et al. (2016) have argued that the three attention networks activate different parts of an extended fronto‐parietal network. The fronto‐parietal network described in the original reports is a larger network and hierarchically organized into separable networks: the ventral attention, dorsal attention, and fronto‐parietal control network (Fox, Corbetta, Snyder, Vincent, & Raichle, 2006; Power et al., 2011; Thomas Yeo et al., 2011; Vincent, Kahn, Snyder, Raichle, & Buckner, 2008). The ventral attention network is thought to support stimulus‐driven bottom‐up attention which is conceptually similar to alerting, the dorsal attention network is thought to support top‐down attention which is conceptually similar to orienting, and the control network is thought to underlie executive functioning and cognitive control which is conceptually similar to the attention control system (Vincent et al., 2008; Vossel, Geng, & Fink, 2014). While our network partition did find three larger networks that involved lateral frontal and posterior parietal cortex, they do not fully match to the three described networks. Our dorsal fronto‐parietal network (IC #14 and #18) corresponds well to the dorsal attention network. Our executive control network (IC #16, #20, and #10) includes dominantly dorsolateral and medial prefrontal cortex and the anterior cingulate and matches the description of the fronto‐parietal control network. Our third fronto‐parietal network (IC #3, #5, and #22), however, matches only partly the description of the ventral attention network. Our network included ventro‐ and dorsolateral, orbitofrontal and frontopolar cortex, as well as inferior parietal and lateral temporal cortex, but did not include the temporo‐parietal junction which represents the major posterior hub in this network Fox et al., 2006; Vossel et al., 2014). In addition to the less optimal correspondence of our fronto‐parietal networks with the three previously described networks, we did not observe a close fit with attention‐evoked activations as well. From the three fronto‐parietal networks in our partition, only the dorsal network showed clear involvement in all attention networks. Single components from the executive control network were involved in orienting and alerting (IC #10, which included mostly superior parietal and posterior cingulate cortex) and control (IC #20 which included mostly dorsolateral and anterior medial prefrontal cortex and the anterior cingulate). The third fronto‐parietal network showed comparatively little involvement in any attention network, with the exception of IC #3, a right lateralized component, that was suppressed during alerting. We therefore conclude that an “extended fronto‐parietal network” does not capture the nature of the three attention networks well. Rather, we see major overlap of the three attention systems within the dorsal fronto‐parietal network and parts of the insular mid‐cingular network. If we would define the “extended fronto‐parietal network” to include these two network components, we would capture the ICN underlying attention, however, we cannot then conclude that the attention systems dissociate within the “extended fronto‐parietal network” but that the “extended fronto‐parietal network” is the attention network.

### 4.2 Orienting: the dorsal and ventral attention network hypothesis

The dorsal and ventral attention network have been proposed as two anatomically and functionally distinct networks (Corbetta & Shulman, 2002). In an attempt to incorporate the dorsal and ventral attention networks into attention network theory, the two networks have been equated to the orienting network (Petersen & Posner, 2012). We will first discuss the representation of the dorsal and ventral attention networks in our ICN partition before discussing their activation by the ANT.

The ventral attention network was initially described as right‐lateralized but later work suggests similar organization in the left hemisphere (Vossel et al., 2014). The ventral attention network features in prominent atlases of canonical ICN (Power et al., 2011; Thomas Yeo et al., 2011) but the labeling as “ventral attention” has been contested. Others have used the labels “salience network” (Seeley et al., 2007) or “cingulo‐opercular network” (Dosenbach, Fair, Cohen, Schlaggar, & Petersen, 2008) to refer to a network with similar anatomy and function. The label ventral attention network has also been used to describe a left‐lateralized network whose implicated brain regions are more suggestive of an involvement in language (Ji et al., 2019; Power et al., 2011). The ventral attention network could represent a right‐lateralized version of the language network (Bernard et al., 2020) but neither we nor others (Ji et al., 2019) have detected a similar right lateralized version of the language network. We decided to follow recent suggestions and use the anatomical label midcingulo‐insular network (Uddin et al., 2019) for three network components that correspond closely to the cingulo‐opercular network in the Cole‐Anticevic and Power‐partition and the ventral attention and salience network in the Yeo‐partition. Of the three components, IC #7 includes bilaterally the tempero‐parietal junction and ventrolateral prefrontal cortex which have been described as hubs in the ventral attention network. The identification of the dorsal attention network in our data was more straightforward. The dorsal attention network is well described in several canonical ICN atlases and we found very consistent correspondence between our components #14 and #18 and the dorsal attention network as described in these atlases.

To assess the correspondence between ICN and the orienting network, we followed previous recommendations and distinguished between different orienting effects: neural activity associated with the disengaging from an invalid spatial cue, the moving and subsequent engaging of the attentional focus to a validly cued spatial location, and the combination of the two (the validity effect) which corresponds to previously described orienting contrasts (Fan et al., 2009; Xuan et al., 2016). Our results confirm the involvement of the dorsal fronto‐parietal and midcingulo‐insular network in orienting. We also observed contributions from a component that we classified as part of an executive control network. This component, however, included many cortical regions that have been ascribed to the dorsal attention network in previous work (Ji et al., 2019; Thomas Yeo et al., 2011). Of note, the component within midcingulo‐insular network that matched most closely the description of the ventral attention network contributed only marginally to the orienting contrasts. While the present results are well in line with the hypothesis by Petersen and Posner (2012) that the orienting network encompasses two networks that correspond to what has been described as dorsal and ventral attention network, it needs to be noted that this relationship is far from specific. We observed similar contributions of the ICN to alerting and control, which does not support the idea of a specific contribution to an anatomically distinct orienting network. Rather, our results indicate that the dorsal fronto‐parietal and midcingulo‐insular network play a domain‐general role in the prioritization of relevant information processing that exceeds a specific contribution to the allocation of attentional resources in space.

### 4.3 Attentional control: the fronto‐parietal cingulo‐opercular hypothesis

Attention network theory assumes an attention control system that is involved in the detection of targets for focal and conscious processing (Posner & Petersen, 1990), guided and controlled visual search (Posner & Dehaene, 1994), and the selection of relevant over distracting information (Fan et al., 2002). Ongoing control involves the maintenance of task‐sets that set the context for moment‐to‐moment adjustments of cognitive processing: Previous studies indicate that set‐maintenance is supported by the cingulo‐opercular network and adjustments of the attentional focus is carried out by the fronto‐parietal network (Dosenbach et al., 2007, 2008). This has led to the proposal that the attention control network relies on these two separate ICN: A fronto‐parietal network that is distinct from the dorsal attention network and the cingulo‐opercular network that we labeled midcingulo‐insular network (Petersen & Posner, 2012). While our data confirm that parts of the midcingulo‐insular network were recruited by the flanker contrast, we saw the strongest contributions from frontal and parietal regions that belonged to the dorsal fronto‐parietal network. From the other two ICN with fronto‐parietal involvement, only one component classified into the executive control network showed additional contribution. This component included dorsolateral prefrontal and anterior cingulate cortex, key regions that have been unequivocally ascribed to the attention control system (Fan et al., 2005; Fan & Posner, 2004; Petersen & Posner, 2012). While our present results are thus consistent with previous findings, they cast doubt that attentional control relies solely on a fronto‐parietal network distinct from the dorsal network and the midcingulo‐insular network. Rather, attentional control seems to be implemented by the dorsal attention network with additional contribution from lateral prefrontal and anterior cingulate cortex. We also observed strong deactivations of the default mode network during attentional control. The traditional view of the default mode network is that of a task‐negative network that stands in an antagonistic relationship with fronto‐parietal networks and de‐activates unspecifically in demanding tasks (Fox, Zhang, Snyder, & Raichle, 2009; Raichle et al., 2001; Shulman et al., 1997). Newer evidence, however, suggests that deactivations in the default mode network encode spatial vision (Szinte & Knapen, 2020) which opens the possibility that the default mode network plays a more direct role in visual attention than expected. Future work is needed to address this hypothesis, but for now we content that the default mode network also contributes to the attentional control network.

### 4.4 Alerting and the midcingulo‐insular network

Alerting refers to a state of increased sensitivity to incoming stimuli (Posner, 2008). In addition to tonic alertness as a self‐initiated state of sustained vigilance, phasic alertness can use external cues to temporarily increase vigilance in anticipation of upcoming information. While both types of alertness are thought to be realized by the same alerting network, the typical alerting contrast in the ANT uses temporal cues to induce a state of alertness and thus taps primarily into the phasic component. The midcingulo‐insular network has been shown to increase its activity and functional connectivity in a task that required tonic alertness (Sadaghiani & D'Esposito, 2014) and increased prestimulus activity in the midcingulo‐insular network leads to faster responses to unpredictable stimuli (Coste & Kleinschmidt, 2016). The midcingulo‐insular network also activates in reaction to rare oddball stimuli, which implies a similar involvement in phasic alerting (Kim, 2014). We subsumed three distinct components under the midcingulo‐insular network and found one of these components to be strongly involved in the alerting contrast, supporting these previous observations. But while we do find evidence for the alerting‐midcingulo‐insular network hypothesis, we cannot conclude that this network is specifically involved in the alerting component of attention. The ICN was consistently recruited by all attention networks and we found widespread activation overlap in the premotor part of the ICN (see Figure 5). The other two components of the midcingulo‐insular network, encompassing either lateral frontal, inferior parietal, and the tempero‐parietal junction or superior parietal and mid‐cingulate cortices did not contribute to alerting. Additionally to the midcingulo‐opercular network we found that alerting activated the dorsal attention network and deactivated a fronto‐parietal component including dorsolateral prefrontal and inferior parietal cortex, as well as the default mode network. As much as we confirm the role of the midcingulo‐insular network in alerting, we neither found a one‐to‐one correspondence between this ICN and the alerting network nor did we find evidence for a specific relationship between the midcingulo‐insular network and alerting in the context of other attention systems.

### 4.5 Overlap between attention networks

We found several ICN components that were involved in all three attention networks. While different brain regions within ICN also tend to co‐activate together during tasks (Smith et al., 2009), there would still be chance that the three attention networks proposed by attention network theory dissociate within a given ICN component. But to the contrary, we found major overlap of the attention systems in posterior parietal cortex along the intraparietal sulcus (the dorsal fronto‐parietal network) and in premotor cortex (dorsal fronto‐parietal and the midcingulo‐insular network). Overlapping activations in premotor cortex occurred in three distinct regions: the bilateral frontal eye field, the bilateral area 6v, and the bilateral premotor eye fields. Covert spatial attention, that is, the adjustment of the attentional focus in the absence of overt eye movements, has been tightly linked to the premotor cortex (Moore, Armstrong, & Fallah, 2003; Rizzolatti, Riggio, Dascola, & Umiltá, 1987) and the frontal eye fields have been identified as the neural origin of the “attentional spotlight” that modulates activity in visual areas (Thompson, 2005). The premotor eye field has also been linked to saccadic eye movements, but also to attention, and the integration of hand and eye movements (Amiez & Petrides, 2009; Genon et al., 2018; Neromyliotis & Moschovakis, 2018). Area 6v is an area in superior premotor cortex adjacent to the frontal eye field and delineates from the frontal eye field regarding its myelin content and its response profile to different tasks (Glasser et al., 2016). We found area 6v involved in the midcingulo‐insular network while the frontal and premotor eye fields belonged to the dorsal fronto‐parietal network. The frontal eye fields and surrounding areas have been previously associated with different attention networks (Xuan et al., 2016) but more work is needed to directly contrast the role of the frontal and premotor eye fields and area 6v within attention networks. We believe that the regions play a role in covert spatial attention by adjusting the attentional focus, irrespective of whether it is moved in space, activated in preparation of upcoming stimuli, or tuned to select relevant over irrelevant information. We also found that visual ICNs contributed to all attention contrasts. While visual activations have been reported previously with the ANT (Xuan et al., 2016), attention network theory dissociates the attention networks from stimulus processing areas (Petersen & Posner, 2012). We presume that visual activations are most likely the result of top down modulation by the attention networks; attentional modulation, for instance, has been described as early as area V1 (Luck et al., 1997). However, we cannot rule out that eye movements in reaction to the cues have led to some spurious activations in visual areas. Future work with a modified ANT is needed to dissociate the source and target of attentional modulation in early visual areas.

### 4.6 Methodological considerations

In the following, we are going to address methodological aspects regarding the definition of attention networks, our resting‐state decomposition into ICNs, and the spatial regression approach.

We defined the attention networks as the set of activated grayordinates in different contrasts in the ANT, the standard protocol proposed by the authors of attention network theory (Fan et al., 2009; Fan & Posner, 2004). This decision was motivated by previous work on attention networks (Fan et al., 2002; Xuan et al., 2016). Defining a network solely on task co‐activations, however, is not without criticism. The term “network” is commonly used to describe a set of network nodes including their mutual relationships (Albert & Barabási, 2002). By simply focusing on task‐evoked activations we thus omitted any information on functional interactions within the attention network. Work on task‐evoked whole‐brain functional connectivity changes suggests that task‐activations and task‐connectivity carry different information (Gerchen & Kirsch, 2017) and task‐connectivity can point toward important network nodes that do not show strong activation changes between task conditions (Markett, Jawinski, Kirsch, & Gerchen, 2020). While the current operationalization of attention networks is thus consistent with previous work and aids interpretability in the context of previous findings, future work will want to utilize methods that aim at functional connectivity to map attention networks in more detail. When combined with analytic approaches from network science, such approach can also highlight different roles of brain area in the context of distributed systems (Zink, Lenartowicz, & Markett, 2021). While we kept the definition of attention networks consistent with previous work, we applied a slightly modified statistical model than Xuan et al. (2016) and did not separate the cue from the target stage in separate regressors. The reason for this was the short onset asynchrony between cues and targets, which is common in the ANT. Despite this difference, our model was able to reproduce the activations for the three main attention networks as described in the literature.

Our main focus was a detailed comparison between activation maps from different task contrasts and the topology of ICN. While the general pattern of ICN has been well‐replicated across studies, acquisition protocols, and analytical approaches, it needs to be noted that ICN are statistical abstractions of BOLD fluctuations and the exact number and topology of reconstructed ICN depends on hyperparameter choices and preprocessing strategies. Different modular decompositions of resting‐state time series have been proposed throughout the literature that feature for instance 6 (Dosenbach et al., 2010), 10 (Smith et al., 2009), 7 or 17 (Thomas Yeo et al., 2011), 12 (Ji et al., 2019), or 13 (Power et al., 2011) ICN. In the absence of a universal ground truth, we decided to achieve our own ICN decomposition of the same participants' resting state timeseries. We applied independent component analysis, an established approach that results in consistent and stable ICN maps (Beckmann, DeLuca, Devlin, & Smith, 2005), does not depend on a priori node definitions (Smith et al., 2011), allows for an automated optimization of the model order parameter (Beckmann & Smith, 2004), and most importantly, operates on the grayordinate‐level which makes a direct comparison of ICN maps and task‐activation maps straightforward. Nevertheless, it needs to be pointed out that the exact parameter has a major impact on the results of the spatial regression analysis. We therefore repeated the analysis with a published ICN partition that follows a similar hierarchical network structure (Thomas Yeo et al., 2011). This analysis yielded similar results and supports the main conclusions.

Despite all progress in network neuroscience, the field has yet to agree on a comprehensive list of ICN and their names (Uddin et al., 2019). To a certain extent, the apparent differences between studies might arise from the rather indirect approach to neural activity inherent to functional neuroimaging, and from parameter choices for clustering and community detection. But more importantly, they can also reflect the hierarchical structure of functional interactions in the brain where larger networks delineate into several smaller networks at higher resolution levels (Betzel et al., 2013; Hilgetag & Goulas, 2020; Meunier, Lambiotte, & Bullmore, 2010). The hierarchical nature of ICN was also reflected in our present ICN partition. We found our reconstructed 23 signal components to correspond to nine larger ICN that have all been described in the literature. Importantly, we observed clear sensory (auditory and visual) and motor networks, which is an essential criterion for a valid network parcellation. Independent component analyses allow the different components to overlap.

We probed the relationship between ICNs and the attention network maps through a spatial regression approach as described previously (Gordon et al., 2012). Since we were interested in the spatial covariation of signals across the entire brain, which is expressed in single statistical parameters, no adjustment of the task activation and IC maps for multiple comparison was required and we submitted unthresholded maps to the regression analyses. By analyzing unthresholded maps, we also made use of the full set of grayordinates and included the full range of grayordinate loadings which aids the interpretation of the spatial regressions' beta weights (positive signs indicate recruitment, negative signs indicate suppression). We verified the absence of multicollinearity between IC components, which is not only a prerequisite for the spatial regression analysis but also a confirmation that our ICA‐approach was successful in yielding spatially independent components. It needs to be noted, however, that the present approach assumes static ICNs that persist across task and resting states and are invariant across participants. While these assumptions hold at large (Cole et al., 2014; Smith et al., 2009), there is still ample evidence for subtle yet reliable variation in network structure across tasks, time, and individuals (Cole et al., 2014; Muldoon & Bassett, 2015; Seitzman et al., 2019). We hope that the present comparison between attention networks and the intrinsic network architecture will stipulate more research into the network‐level representation of attention that will extend the current focus to temporal dynamics and individual differences.

Unfortunately, we did not have the technical equipment to record eye gaze data, which is a shortcoming of the present work that needs to be mentioned. Participants were instructed to maintain fixation throughout each trial, to encourage covert shifts of attention. Stimulus display and timing did not require eye movements, but without eye tracking data, there is no direct way to confirm that all participants followed this instruction at all times. While it seems to be possible to infer gaze location from functional MRI directly, relevant software tools had not been publicly available yet (Frey, Nau, & Doeller, 2021).

### 4.7 Conclusions regarding attention network theory

While we found a good overall correspondence between the attention network maps and the brain's intrinsic connectivity architecture, we did not find unique relationships between any attention network maps and single ICN, challenging most previous conjectures on the representation of attention at the network level. Each attention contrasts activated several ICN, and we found that all attention networks converged within the dorsal fronto‐parietal and midcingulo‐opercular network, pointing toward a shared neural resource between the different attention networks. Given that interactions and spatial overlap between attention networks have been described previously (Xuan et al., 2016), we argue to reconsider the notion of separable and independent attention networks. Instead, we propose that attention is supported by a distributed network in which different subroutines of attention (alerting, orienting, and control) segregate into different subnetworks and are integrated by hubs in the dorsal fronto‐parietal and midcingulo‐insular network. While this proposal requires further empirical investigations, it would be well in line with several discoveries regarding the network‐level representation of cognitive control and higher cognition (Braun et al., 2015; Cohen & D'Esposito, 2016; Cohen, Gallen, Jacobs, Lee, & D'Esposito, 2014; Cole et al., 2013; Zink et al., 2021). At the same time, we propose to reconsider terminology: Using the term “network” for the distributed patterns of task‐evoked activations and for distributed patterns of intrinsically generated functional connectivity alike suggests too much of a conceptual equivalence which is not supported by the data.

## CONFLICT OF INTEREST

The authors declare no potential conflict of interest.

## Supporting information


**Appendix S1.** Supporting Information.

## Data Availability

All preprocessing code for structural and functional preprocessing can be obtained from https://github.com/Washington-University/HCPpipelines. Group‐level activation maps and ICN maps (thresholded and unthresholded) and Matlab code to match ICN to templates, run the spatial regression analysis and subsequent analyses are available on the Open Science Framework (https://osf.io/st9ae/).

## References

[hbm25734-bib-0001] Albert, R. , & Barabási, A.‐L. (2002). Statistical mechanics of complex networks. Reviews of Modern Physics, 74(1), 47.

[hbm25734-bib-0002] Amiez, C. , & Petrides, M. (2009). Anatomical organization of the eye fields in the human and non‐human primate frontal cortex. Progress in Neurobiology, 89(2), 220–230. 10.1016/j.pneurobio.2009.07.010 19665515

[hbm25734-bib-0003] Beckmann, C. F. , DeLuca, M. , Devlin, J. T. , & Smith, S. M. (2005). Investigations into resting‐state connectivity using independent component analysis. Philosophical Transactions of the Royal Society B: Biological Sciences, 360(1457), 1001–1013. 10.1002/(SICI)1097-0193(1999)8:2/3<151::AID-HBM13>3.0.CO;2-5 PMC1854918 16087444

[hbm25734-bib-0004] Beckmann, C. F. , & Smith, S. M. (2004). Probabilistic independent component analysis for functional magnetic resonance imaging. IEEE Transactions on Medical Imaging, 23(2), 137–152. 10.1109/TMI.2003.822821 14964560

[hbm25734-bib-0005] Bernard, F. , Lemee, J. , Mazerand, E. , Leiber, L. , Menei, P. , & Ter Minassian, A. (2020). The ventral attention network: The mirror of the language network in the right brain hemisphere. Journal of Anatomy, 237(4), 632–642. 10.1111/joa.13223 32579719 PMC7495290

[hbm25734-bib-0006] Betzel, R. F. , Griffa, A. , Avena‐Koenigsberger, A. , Goñi, J. , Thiran, J.‐P. , Hagmann, P. , & Sporns, O. (2013). Multi‐scale community organization of the human structural connectome and its relationship with resting‐state functional connectivity. Network Science, 1(3), 353–373. 10.1017/nws.2013.19

[hbm25734-bib-0007] Braun, U. , Schäfer, A. , Walter, H. , Erk, S. , Romanczuk‐Seiferth, N. , Haddad, L. , … Bassett, D. S. (2015). Dynamic reconfiguration of frontal brain networks during executive cognition in humans. Proceedings of the National Academy of Sciences, 112(37), 11678–11683. 10.1073/pnas.1422487112 PMC4577153 26324898

[hbm25734-bib-0008] Brefczynski, J. A. , & DeYoe, E. A. (1999). A physiological correlate of the “spotlight” of visual attention. Nature Neuroscience, 2(4), 370–374. 10.1038/7280 10204545

[hbm25734-bib-0009] Callejas, A. , Lupiáñez, J. , & Tudela, P. (2004). The three attentional networks: On their independence and interactions. Brain and Cognition, 54(3), 225–227. 10.1016/j.bandc.2004.02.012 15050779

[hbm25734-bib-0010] Cohen, J. R. , & D'Esposito, M. (2016). The segregation and integration of distinct brain networks and their relationship to cognition. Journal of Neuroscience, 36(48), 12083–12094. 10.1523/JNEUROSCI.2965-15.2016 27903719 PMC5148214

[hbm25734-bib-0011] Cohen, J. R. , Gallen, C. L. , Jacobs, E. G. , Lee, T. G. , & D'Esposito, M. (2014). Quantifying the reconfiguration of intrinsic networks during working memory. PLoS One, 9(9), e106636. 10.1371/journal.pone.0106636 25191704 PMC4156328

[hbm25734-bib-0012] Cole, M. W. , Bassett, D. S. , Power, J. D. , Braver, T. S. , & Petersen, S. E. (2014). Intrinsic and task‐evoked network architectures of the human brain. Neuron, 83(1), 238–251. 10.1016/j.neuron.2014.05.014 24991964 PMC4082806

[hbm25734-bib-0013] Cole, M. W. , Reynolds, J. R. , Power, J. D. , Repovs, G. , Anticevic, A. , & Braver, T. S. (2013). Multi‐task connectivity reveals flexible hubs for adaptive task control. Nature Publishing Group, 16(9), 1348–1355. 10.1038/nn.3470 PMC3758404 23892552

[hbm25734-bib-0014] Corbetta, M. , & Shulman, G. L. (2002). Control of goal‐oriented and stimulus‐driven attention in the brain. Nature Reviews Neuroscience, 3(3), 215–229. 10.1038/nrn755 11994752

[hbm25734-bib-0015] Coste, C. P. , & Kleinschmidt, A. (2016). Cingulo‐opercular network activity maintains alertness. NeuroImage, 128, 264–272. 10.1016/j.neuroimage.2016.01.026 26801604

[hbm25734-bib-0016] Cowan, N. (1999). An embedded‐processes model of working memory. In A. Miyake & S. Priti (Eds.), Models of working memory: Mechanisms of active maintenance and executive control (pp. 62–101). Cambridge: Cambridge University Press.

[hbm25734-bib-0017] Dosenbach, N. U. F. , Fair, D. A. , Cohen, A. L. , Schlaggar, B. L. , & Petersen, S. E. (2008). A dual‐networks architecture of top‐down control. Trends in Cognitive Sciences, 12(3), 99–105. 10.1016/j.tics.2008.01.001 18262825 PMC3632449

[hbm25734-bib-0018] Dosenbach, N. U. F. , Fair, D. A. , Miezin, F. M. , Cohen, A. L. , Wenger, K. K. , Dosenbach, R. A. T. , … Petersen, S. E. (2007). Distinct brain networks for adaptive and stable task control in humans. Proceedings of the National Academy of Sciences, 104(26), 11073–11078. 10.1073/pnas.0704320104 PMC1904171 17576922

[hbm25734-bib-0019] Dosenbach, N. U. F. , Nardos, B. , Cohen, A. L. , Fair, D. A. , Power, J. D. , Church, J. A. , … Schlaggar, B. L. (2010). Prediction of individual brain maturity using fMRI. Science (New York, N.Y.), 329(5997), 1358–1361. 10.1126/science.1194144 PMC3135376 20829489

[hbm25734-bib-0020] Egner, T. , & Hirsch, J. (2005). Cognitive control mechanisms resolve conflict through cortical amplification of task‐relevant information. Nature Neuroscience, 8(12), 1784–1790. 10.1038/nn1594 16286928

[hbm25734-bib-0021] Eriksen, B. A. , & Eriksen, C. W. (1974). Effects of noise letters upon the identification of a target letter in a nonsearch task. Perception & Psychophysics, 16(1), 143–149. 10.3758/BF03203267

[hbm25734-bib-0022] Fan, J. , Gu, X. , Guise, K. G. , Liu, X. , Fossella, J. , Wang, H. , & Posner, M. I. (2009). Testing the behavioral interaction and integration of attentional networks. Brain and Cognition, 70(2), 209–220. 10.1016/j.bandc.2009.02.002 19269079 PMC2674119

[hbm25734-bib-0023] Fan, J. , Mccandliss, B. , Fossella, J. , Flombaum, J. , & Posner, M. (2005). The activation of attentional networks. NeuroImage, 26(2), 471–479. 10.1016/j.neuroimage.2005.02.004 15907304

[hbm25734-bib-0024] Fan, J. , McCandliss, B. D. , Sommer, T. , Raz, A. , & Posner, M. I. (2002). Testing the efficiency and independence of attentional networks. Journal of Cognitive Neuroscience, 14(3), 340–347. 10.1162/089892902317361886 11970796

[hbm25734-bib-0025] Fan, J. , & Posner, M. (2004). Human Attentional networks. Psychiatrische Praxis, 31, 210–214. 10.1055/s-2004-828484 15586312

[hbm25734-bib-0026] Fan, J. , Wu, Y. , Fossella, J. A. , & Posner, M. I. (2001). Assessing the heritability of attentional networks. BMC Neuroscience, 7. 10.1186/1471-2202-2-14 PMC57000 11580865

[hbm25734-bib-0027] Fossella, J. , Sommer, T. , Fan, J. , Wu, Y. , Swanson, J. M. , Pfaff, D. W. , & Posner, M. I. (2002). Assessing the molecular genetics of attention networks. BMC Neuroscience, 3(1), 14. 10.1186/1471-2202-3-14 12366871 PMC130047

[hbm25734-bib-0028] Fox, M. D. , Corbetta, M. , Snyder, A. Z. , Vincent, J. L. , & Raichle, M. E. (2006). Spontaneous neuronal activity distinguishes human dorsal and ventral attention systems. Proceedings of the National Academy of Sciences, 103(26), 10046–10051. 10.1073/pnas.0604187103 PMC1480402 16788060

[hbm25734-bib-0029] Fox, M. D. , & Raichle, M. E. (2007). Spontaneous fluctuations in brain activity observed with functional magnetic resonance imaging. Nature Reviews Neuroscience, 8(9), 700–711. 10.1038/nrn2201 17704812

[hbm25734-bib-0030] Fox, M. D. , Zhang, D. , Snyder, A. Z. , & Raichle, M. E. (2009). The global signal and observed anticorrelated resting state brain networks. Journal of Neurophysiology, 101(6), 3270–3283. 10.1152/jn.90777.2008 19339462 PMC2694109

[hbm25734-bib-0031] Frey, M. , Nau, M. , & Doeller, C. F. (2021). Magnetic resonance‐based eye tracking using deep neural networks. Natur Neuroscience, 24, 1772–1779. 10.1101/2020.11.30.401323 34750593 PMC10097595

[hbm25734-bib-0032] Genon, S. , Reid, A. , Li, H. , Fan, L. , Müller, V. I. , Cieslik, E. C. , … Eickhoff, S. B. (2018). The heterogeneity of the left dorsal premotor cortex evidenced by multimodal connectivity‐based parcellation and functional characterization. NeuroImage, 170, 400–411. 10.1016/j.neuroimage.2017.02.034 28213119 PMC5555826

[hbm25734-bib-0033] Gerchen, M. F. , & Kirsch, P. (2017). Combining task‐related activation and connectivity analysis of fMRI data reveals complex modulation of brain networks: Complex task modulation of brain networks. Human Brain Mapping, 38, 5726–5739. 10.1002/hbm.23762 28782871 PMC6866920

[hbm25734-bib-0034] Glasser, M. F. , Smith, S. M. , Marcus, D. S. , Andersson, J. L. R. , Auerbach, E. J. , Behrens, T. E. J. , … Van Essen, D. C. (2016). The Human Connectome Project's neuroimaging approach. Nature Neuroscience, 19(9), 1175–1187. 10.1038/nn.4361 27571196 PMC6172654

[hbm25734-bib-0035] Glasser, M. F. , Sotiropoulos, S. N. , Wilson, J. A. , Coalson, T. S. , Fischl, B. , Andersson, J. L. , … WU‐Minn HCP Consortium . (2013). The minimal preprocessing pipelines for the Human Connectome Project. NeuroImage, 80(C, 105–124. 10.1016/j.neuroimage.2013.04.127 23668970 PMC3720813

[hbm25734-bib-0036] Gordon, E. M. , Stollstorff, M. , & Vaidya, C. J. (2012). Using spatial multiple regression to identify intrinsic connectivity networks involved in working memory performance. Human Brain Mapping, 33(7), 1536–1552. 10.1002/hbm.21306 21761505 PMC3374884

[hbm25734-bib-0037] Guillaume, B. , Hua, X. , Thompson, P. M. , Waldorp, L. , & Nichols, T. E. (2014). Fast and accurate modelling of longitudinal and repeated measures neuroimaging data. NeuroImage, 94, 287–302. 10.1016/j.neuroimage.2014.03.029 24650594 PMC4073654

[hbm25734-bib-0038] Harms, M. P. , Somerville, L. H. , Ances, B. M. , Andersson, J. , Barch, D. M. , Bastiani, M. , … Yacoub, E. (2018). Extending the Human Connectome Project across ages: Imaging protocols for the lifespan development and aging projects. NeuroImage, 183, 972–984. 10.1016/j.neuroimage.2018.09.060 30261308 PMC6484842

[hbm25734-bib-0039] Heinze, H. J. , Mangun, G. R. , Burchert, W. , Hinrichs, H. , Scholz, M. , Münte, T. F. , … Hillyard, S. A. (1994). Combined spatial and temporal imaging of brain activity during visual selective attention in humans. Nature, 372(6506), 543–546. 10.1038/372543a0 7990926

[hbm25734-bib-0040] Hilgetag, C. C. , & Goulas, A. (2020). ‘Hierarchy’ in the organization of brain networks. Philosophical Transactions of the Royal Society B: Biological Sciences, 375(1796), 20190319. 10.1098/rstb.2019.0319 PMC7061955 32089116

[hbm25734-bib-0041] Ji, J. L. , Spronk, M. , Kulkarni, K. , Repovš, G. , Anticevic, A. , & Cole, M. W. (2019). Mapping the human brain's cortical‐subcortical functional network organization. NeuroImage, 185, 35–57. 10.1016/j.neuroimage.2018.10.006 30291974 PMC6289683

[hbm25734-bib-0042] Kastner, S. , Pinsk, M. A. , De Weerd, P. , Desimone, R. , & Ungerleider, L. G. (1999). Increased activity in human visual cortex during directed attention in the absence of visual stimulation. Neuron, 22(4), 751–761. 10.1016/S0896-6273(00)80734-5 10230795

[hbm25734-bib-0043] Kim, H. (2014). Involvement of the dorsal and ventral attention networks in oddball stimulus processing: A meta‐analysis. Human Brain Mapping, 35(5), 2265–2284. 10.1002/hbm.22326 23900833 PMC6868981

[hbm25734-bib-0044] Luck, S. J. , Chelazzi, L. , Hillyard, S. A. , & Desimone, R. (1997). Neural mechanisms of spatial selective attention in areas V1, V2, and V4 of macaque visual cortex. Journal of Neurophysiology, 77(1), 24–42. 10.1152/jn.1997.77.1.24 9120566

[hbm25734-bib-0045] Markett, S. , Jawinski, P. , Kirsch, P. , & Gerchen, M. F. (2020). Specific and segregated changes to the functional connectome evoked by the processing of emotional faces: A task‐based connectome study. Scientific Reports, 10(1), 4822. 10.1038/s41598-020-61522-0 PMC7076018 32179856

[hbm25734-bib-0046] Markett, S. , Reuter, M. , Montag, C. , Voigt, G. , Lachmann, B. , Rudorf, S. , … Weber, B. (2014). Assessing the function of the fronto‐parietal attention network: Insights from resting‐state fMRI and the attentional network test: Assessing the function of the fronto‐parietal attention network. Human Brain Mapping, 35(4), 1700–1709. 10.1002/hbm.22285 23670989 PMC6869384

[hbm25734-bib-0047] Meunier, D. , Lambiotte, R. , & Bullmore, E. T. (2010). Modular and hierarchically modular organization of brain networks. Frontiers in Neuroscience, 4, 200. 10.3389/fnins.2010.00200 21151783 PMC3000003

[hbm25734-bib-0048] Moore, T. , Armstrong, K. M. , & Fallah, M. (2003). Visuomotor origins of covert spatial attention. Neuron, 40(4), 671–683. 10.1016/S0896-6273(03)00716-5 14622573

[hbm25734-bib-0049] Muldoon, S. F. , & Bassett, D. S. (2016). Network and multilayer network approaches to understanding human brain dynamics. Philosophy of Science, 83(5), 710–720. 10.1086/687857

[hbm25734-bib-0050] Müller, N. G. , Bartelt, O. A. , Donner, T. H. , Villringer, A. , & Brandt, S. A. (2003). A physiological correlate of the “zoom lens” of visual attention. The Journal of Neuroscience, 23(9), 3561–3565. 10.1523/JNEUROSCI.23-09-03561.2003 12736325 PMC6742201

[hbm25734-bib-0051] Neromyliotis, E. , & Moschovakis, A. K. (2018). Response properties of saccade‐related neurons of the post‐arcuate premotor cortex. Journal of Neurophysiology, 119(6), 2291–2306. 10.1152/jn.00669.2017 29537912

[hbm25734-bib-0052] Nickerson, L. D. (2018). Replication of resting state‐task network correspondence and novel findings on brain network activation during task fMRI in the human Connectome project study. Scientific Reports, 8(17543). 10.1038/s41598-018-35209-6 PMC6277426 30510165

[hbm25734-bib-0053] O'Craven, K. M. , Rosen, B. R. , Kwong, K. K. , Treisman, A. , & Savoy, R. L. (1997). Voluntary attention modulates fMRI activity in human MT–MST. Neuron, 18(4), 591–598. 10.1016/S0896-6273(00)80300-1 9136768

[hbm25734-bib-0054] Petersen, S. E. , & Posner, M. I. (2012). The attention system of the human brain: 20 years after. Annual Review of Neuroscience, 35, 73–89. 10.1146/annurev-neuro-062111-150525 PMC3413263 22524787

[hbm25734-bib-0055] Posner, M. I. (1980). Orienting of attention. Quarterly Journal of Experimental Psychology, 32(1), 3–25. 10.1080/00335558008248231 7367577

[hbm25734-bib-0056] Posner, M. I. (2008). Measuring alertness. Annals of the New York Academy of Sciences, 1129(1), 193–199. 10.1196/annals.1417.011 18591480

[hbm25734-bib-0057] Posner, M. I. , & Dehaene, S. (1994). Attentional networks. Trends in Neurosciences, 17(2), 75–79. 7512772 10.1016/0166-2236(94)90078-7

[hbm25734-bib-0058] Posner, M. I. , & Fan, J. (2008). Attention as an organ system. In J Pomerantz , (ed.), Topics in Integrative Neuroscience (pp. 31–61). Cambridge: Cambridge University Press.

[hbm25734-bib-0059] Posner, M. I. , & Petersen, S. E. (1990). The attention system of the human brain. Annual Review of Neuroscience, 13(1), 25–42. 10.1146/annurev.ne.13.030190.000325 2183676

[hbm25734-bib-0060] Posner, M. I. , & Rothbart, M. K. (2007). Research on attention networks as a model for the integration of psychological science. Annual Review of Psychology, 58(1), 1–23. 10.1146/annurev.psych.58.110405.085516 17029565

[hbm25734-bib-0061] Power, J. D. , Cohen, A. L. , Nelson, S. M. , Wig, G. S. , Barnes, K. A. , Church, J. A. , … Petersen, S. E. (2011). Functional network organization of the human brain. Neuron, 72(4), 665–678. 10.1016/j.neuron.2011.09.006 22099467 PMC3222858

[hbm25734-bib-0062] Raichle, M. E. , MacLeod, A. M. , Snyder, A. Z. , Powers, W. J. , Gusnard, D. A. , & Shulman, G. L. (2001). A default mode of brain function. Proceedings of the National Academy of Sciences of the United States of America, 98(2), 676–682. 10.1073/pnas.98.2.676 11209064 PMC14647

[hbm25734-bib-0063] Rees, G. , Frith, C. D. , & Lavie, N. (1997). Modulating irrelevant motion perception by varying attentional load in an unrelated task. Science, 278(5343), 1616–1619. 10.1126/science.278.5343.1616 9374459

[hbm25734-bib-0064] Reuter, M. , Ott, U. , Vaitl, D. , & Hennig, J. (2007). Impaired executive control is associated with a variation in the promoter region of the tryptophan hydroxylase 2 gene. Journal of Cognitive Neuroscience, 19(3), 401–408. 10.1162/jocn.2007.19.3.401 17335389

[hbm25734-bib-0065] Rizzolatti, G. , Riggio, L. , Dascola, I. , & Umiltá, C. (1987). Reorienting attention across the horizontal and vertical meridians: Evidence in favor of a premotor theory of attention. Neuropsychologia, 25(1), 31–40. 10.1016/0028-3932(87)90041-8 3574648

[hbm25734-bib-0066] Robinson, E. C. , Garcia, K. , Glasser, M. F. , Chen, Z. , Coalson, T. S. , Makropoulos, A. , … Rueckert, D. (2018). Multimodal surface matching with higher‐order smoothness constraints. NeuroImage, 167, 453–465. 10.1016/j.neuroimage.2017.10.037 29100940 PMC5991912

[hbm25734-bib-0067] Sadaghiani, S. , & D'Esposito, M. (2014). Functional characterization of the cingulo‐opercular network in the maintenance of tonic alertness. Cerebral Cortex, 25(9), 2763–2773. 10.1093/cercor/bhu072 24770711 PMC4537431

[hbm25734-bib-0068] Seeley, W. W. , Menon, V. , Schatzberg, A. F. , Keller, J. , Glover, G. H. , Kenna, H. , … Greicius, M. D. (2007). Dissociable intrinsic connectivity networks for salience processing and executive control. Journal of Neuroscience, 27(9), 2349–2356. 10.1523/JNEUROSCI.5587-06.2007 17329432 PMC2680293

[hbm25734-bib-0069] Seitzman, B. A. , Gratton, C. , Laumann, T. O. , Gordon, E. M. , Adeyemo, B. , Dworetsky, A. , … Petersen, S. E. (2019). Trait‐like variants in human functional brain networks. Proceedings of the National Academy of Sciences, 116(45), 22851–22861. 10.1073/pnas.1902932116 PMC6842602 31611415

[hbm25734-bib-0070] Shulman, G. L. , Fiez, J. A. , Corbetta, M. , Buckner, R. L. , Miezin, F. M. , Raichle, M. E. , & Petersen, S. E. (1997). Common blood flow changes across visual tasks: II. Decreases in cerebral cortex. Journal of Cognitive Neuroscience, 9(5), 648–663. 10.1162/jocn.1997.9.5.648 23965122

[hbm25734-bib-0071] Smith, S. M. , Fox, P. T. , Miller, K. L. , Glahn, D. C. , Fox, P. M. , Mackay, C. E. , … Beckmann, C. F. (2009). Correspondence of the brain's functional architecture during activation and rest. Proceedings of the National Academy of Sciences of the United States of America, 106(31), 13040–13045. 10.1073/pnas.0905267106 19620724 PMC2722273

[hbm25734-bib-0072] Smith, S. M. , Miller, K. L. , Salimi‐Khorshidi, G. , Webster, M. , Beckmann, C. F. , Nichols, T. E. , … Woolrich, M. W. (2011). Network modelling methods for fMRI. NeuroImage, 54(2), 875–891. 10.1016/j.neuroimage.2010.08.063 20817103

[hbm25734-bib-0073] Smith, S. M. , Vidaurre, D. , Beckmann, C. F. , Glasser, M. F. , Jenkinson, M. , Miller, K. L. , … Van Essen, D. C. (2013). Functional connectomics from resting‐state fMRI. Trends in Cognitive Sciences, 17(12), 666–682. 10.1016/j.tics.2013.09.016 24238796 PMC4004765

[hbm25734-bib-0074] Szinte, M. , & Knapen, T. (2020). Visual organization of the default network. Cerebral Cortex, 30(6), 3518–3527. 10.1093/cercor/bhz323 32031204 PMC7232993

[hbm25734-bib-0075] Thomas Yeo, B. T. , Krienen, F. M. , Sepulcre, J. , Sabuncu, M. R. , Lashkari, D. , Hollinshead, M. , … Buckner, R. L. (2011). The organization of the human cerebral cortex estimated by intrinsic functional connectivity. Journal of Neurophysiology, 106(3), 1125–1165. 10.1152/jn.00338.2011 21653723 PMC3174820

[hbm25734-bib-0076] Thompson, K. G. (2005). Neuronal basis of covert spatial attention in the frontal eye field. Journal of Neuroscience, 25(41), 9479–9487. 10.1523/JNEUROSCI.0741-05.2005 16221858 PMC2804969

[hbm25734-bib-1001] Toro, R. , Fox, P. T. , & Paus, T. (2008). Functional coactivation map of the human brain. Cerebral Cortex, 18(11), 2553–2559. 10.1093/cercor/bhn014 18296434 PMC2567424

[hbm25734-bib-0077] Uddin, L. Q. , Yeo, B. T. T. , & Spreng, R. N. (2019). Towards a universal taxonomy of macro‐scale functional human brain networks. Brain Topography, 32(6), 926–942. 10.1007/s10548-019-00744-6 31707621 PMC7325607

[hbm25734-bib-0078] van den Heuvel, M. P. , & Hulshoff Pol, H. E. (2010). Exploring the brain network: A review on resting‐state fMRI functional connectivity. European Neuropsychopharmacology, 20(8), 519–534. 10.1016/j.euroneuro.2010.03.008 20471808

[hbm25734-bib-0079] Vincent, J. L. , Kahn, I. , Snyder, A. Z. , Raichle, M. E. , & Buckner, R. L. (2008). Evidence for a frontoparietal control system revealed by intrinsic functional connectivity. Journal of Neurophysiology, 100(6), 3328–3342. 10.1152/jn.90355.2008 18799601 PMC2604839

[hbm25734-bib-0080] Visintin, E. , De Panfilis, C. , Antonucci, C. , Capecci, C. , Marchesi, C. , & Sambataro, F. (2015). Parsing the intrinsic networks underlying attention: A resting state study. Behavioural Brain Research, 278, 315–322. 10.1016/j.bbr.2014.10.002 25311282

[hbm25734-bib-0081] Vossel, S. , Geng, J. J. , & Fink, G. R. (2014). Dorsal and ventral attention systems. The Neuroscientist, 20(2), 150–159. 10.1177/1073858413494269 23835449 PMC4107817

[hbm25734-bib-0082] Xuan, B. , Mackie, M.‐A. , Spagna, A. , Wu, T. , Tian, Y. , Hof, P. R. , & Fan, J. (2016). The activation of interactive attentional networks. NeuroImage, 129, 308–319. 10.1016/j.neuroimage.2016.01.017 26794640 PMC4803523

[hbm25734-bib-0083] Zink, N. , Lenartowicz, A. , & Markett, S. (2021). A new era for executive function research: On the transition from centralized to distributed executive functioning. Neuroscience & Biobehavioral Reviews, 124, 235–244. 10.1016/j.neubiorev.2021.02.011 33582233 PMC8420078

