# Supplementary material for: Attention networks and the intrinsic network structure of the human brain
Source: Hum Brain Mapp. 2021 Dec 9;43(4):1431–48. doi: 10.1002/hbm.25734 (PMC8837576; doi:10.1002/hbm.25734)
Supplement: Supplementary file 1 — Appendix S1. Supporting Information. [file HBM-43-1431-s001.docx]

Supplementary Information

Attention Networks and the Intrinsic Network Structure of the Human Brain

Sebastian Markett^1*^, David Nothdurfter^1^, Antonia Focsa^1^, Martin Reuter^2^, Philippe Jawinski^1^

**T1. Comparison of ICA components and resting-state networks from other parcellations.**

We compared each IC component to existing network parcellations by binarizing the map at z>|3| and computing the spatial overlap with existing templates via the Jakkard index (Steen et al., 2011). Comparison with the cortical and subcortical Cole-Anticevic parcellation were done at the grayordinate-level. For the other three atlases, we converted cifti dscalar to giftis prior to comparison. The table gives the template network with the highest spatial overlap. Boldface indicates a consistent labeling across multiple parcellations.

Table T1 Spatial comparison between our ICA decomposition and existing network atlases.

|  |  | Cole-Anticevic | Yeo 7 | Yeo 17 | Power |
| --- | --- | --- | --- | --- | --- |
| red | ICN #16 | Frontoparietal | Default | Default A | Default |
|  | ICN #20 | Cingulo-Opercular | FrontoParietal | SalVentAttnB | Default |
|  | ICN #10 | DorsalAttn | DorsalAttn | Default C | Default |
|  | ICN #3 | **Frontoparietal** | **FrontoParietal** | **Control B** | Default |
|  | ICN #5 | **Frontoparietal** | **FrontoParietal** | **Control A** | **FrontoParietal** |
|  | ICN #22 | **Frontoparietal** | **FrontoParietal** | **Control B** | **FrontoParietal** |
| magenta | ICN #2 | **Default** | **Default** | **Default B** | **Default** |
|  | ICN #4 | **Default** | **Default** | **Default A** | **Default** |
|  | ICN #11 | **Default** | **Default** | **Default B** | **Default** |
|  | ICN #9 | **Language** | Default | Default B | VentralAttn |
| light green | ICN #1 | **Visual2** | **Visual** | **VisualCent** | **Visual** |
|  | ICN #19 | **Visual2** | **Visual** | **VisualCent** | **Visual** |
|  | ICN #6 | **Visual1** | **Visual** | **VisualPeri** | **Visual** |
|  | ICN #17 | **Visual2** | **Visual** | **VisualCent** | **Visual** |
| blue | ICN #7 | Cingulo-Opercular | FrontoParietal | SalVentAttnB | FrontoParietal |
|  | ICN #12 | Cingulo-Opercular | VentralAttn | SalVentAttnA | CinguloOpercular |
|  | ICN #8 | SM | VentralAttn | SalVentAttnA | SM "Hand" |
|  | ICN #14 | **DorsalAttn** | **DorsalAttn** | Control A | FrontoParietal |
|  | ICN #18 | **DorsalAttn** | **DorsalAttn** | **DorsAttnB** | **DorsalAttn** |
| green | ICN #13 | **SM** | **SM** | **SM B** | **SM "Mouth"** |
|  | ICN #15 | **SM** | **SM** | **SM A** | **SM "Hand"** |
|  | ICN #21 | **SM** | **SM** | **SM A** | **SM "Hand"** |
|  | ICN #23 | **Auditory** | SM | SM B | **Auditory** |

**S1. Comparison of IC components and the Glasser multimodal parcellation**

We used the Glasser cortical parcellation and the corresponding mapping of the 360 cortical regions to 22 cortices to annotate the 23 ICN. The 23 ICN maps and the Glasser parcellation were converted to gifti files (one per hemisphere). The ICN maps were thresholded at Z>|4|. We then calculated for each cortex (e.g. early visual cortex) the percentage of cortical parcels that overlapped with each thresholded ICN. Results are plotted in figure S1.


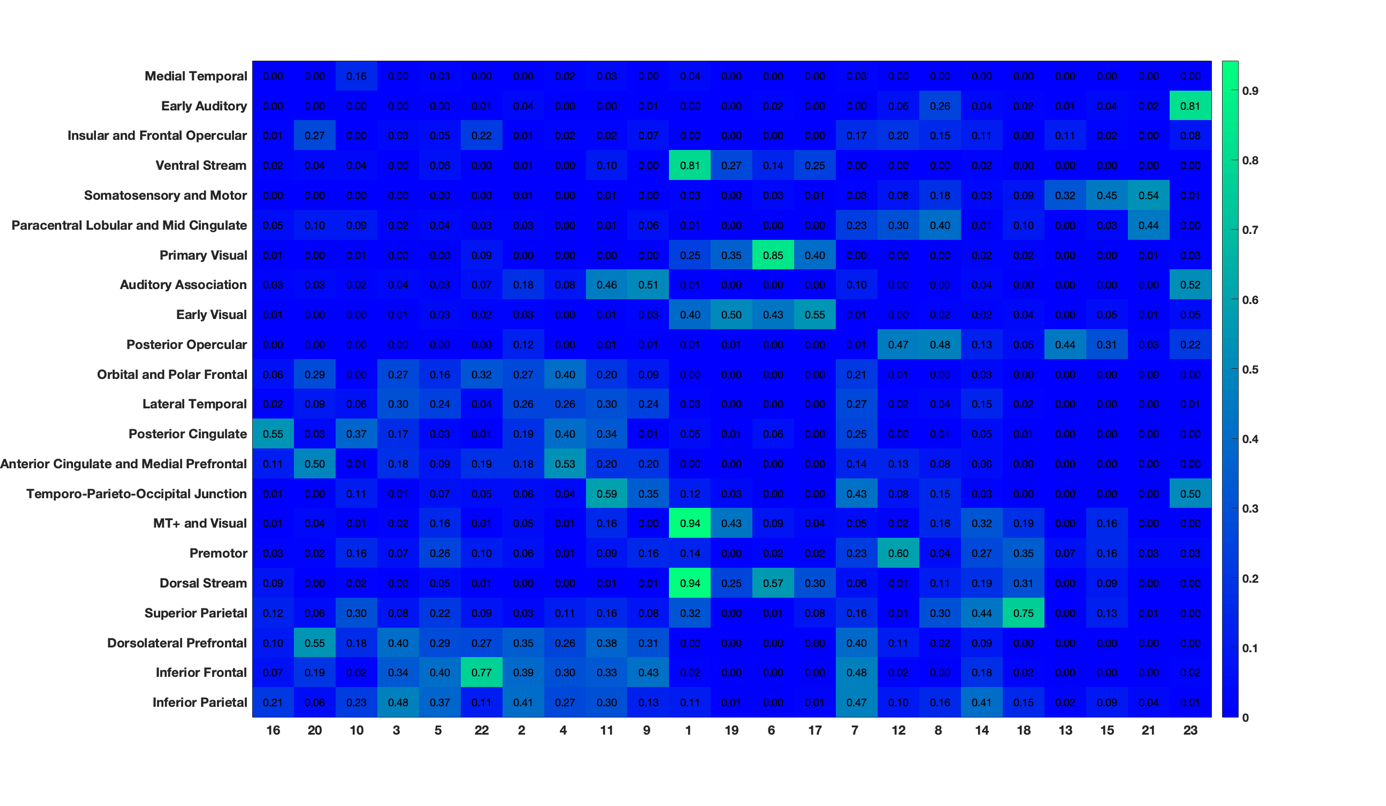


Figure S1: Overlap between ICN components and the 22 cortices (groups of cortical areas) from the multimodal parcellation.

**S2: Collinearity diagnostics**

We present a tableplot (Friendly et al., 2009) with condition indices and variance decomposition proportions for each regressor (i.e. ICA component): Condition indices (the square root of the ratio of the maximum eigenvalue to each eigenvalue from the correlation matrix between standardized predictor variables) is given in the first column. By convention, condition indices <5 are considered unproblematic. The other columns give variance decomposition proportions that show the contribution of each predictor to potential variance inflation. Convention regards a predictor with two or more variance proportions >.5 as collinear. For sake of simplicity, all predictors are labeled X1, …, XN. Neither collinearity diagnostic indicated collinear relationships among predictors.

Friendly, M., & Kwan, E. (2009). Where’s Waldo? Visualizing Collinearity Diagnostics. *The* *American Statistician, 63(1),* 56–65. https://doi.org/10.1198/tast.2009.0012

Figure S2: Collinearity Diagnostics for 23 predictors (ICA components).

**S3. Volumetric activation maps and region of interest masks**

We assessed correspondence of our activation maps with previously reported voxel locations by creating binary masks consisting of spheres (8mm) around the peak coordinates reported by Xuan et al. (2016) for all five ANT contrasts (see Xuan et al. 2016, NeuroImage, Tables 2-4). We extracted parameters from individua contrasts images and ran a one-sample t-test against zero for each attention contrast. Because a mapping of volumetric voxel locations to the surface is not straightforward, we performed the comparisons with volumetric data. All first-level models were run exactly as described in the main text with the difference that we did not use ‘fake-niftis’ based on cifti-grayordinates with 2mm surface smoothing but volumetric niftis with 4 mm volumetric smoothing. For display purposes, we ran 2^nd^ level models (one-sample ttests) in SPM12. Maps are thresholded at p<.05, FEW-corrected at the cluster level with a cluster defining threshold of p<.01, uncorrected (see main text and Xuan et al., 2016) for a justification of the parameters.

**
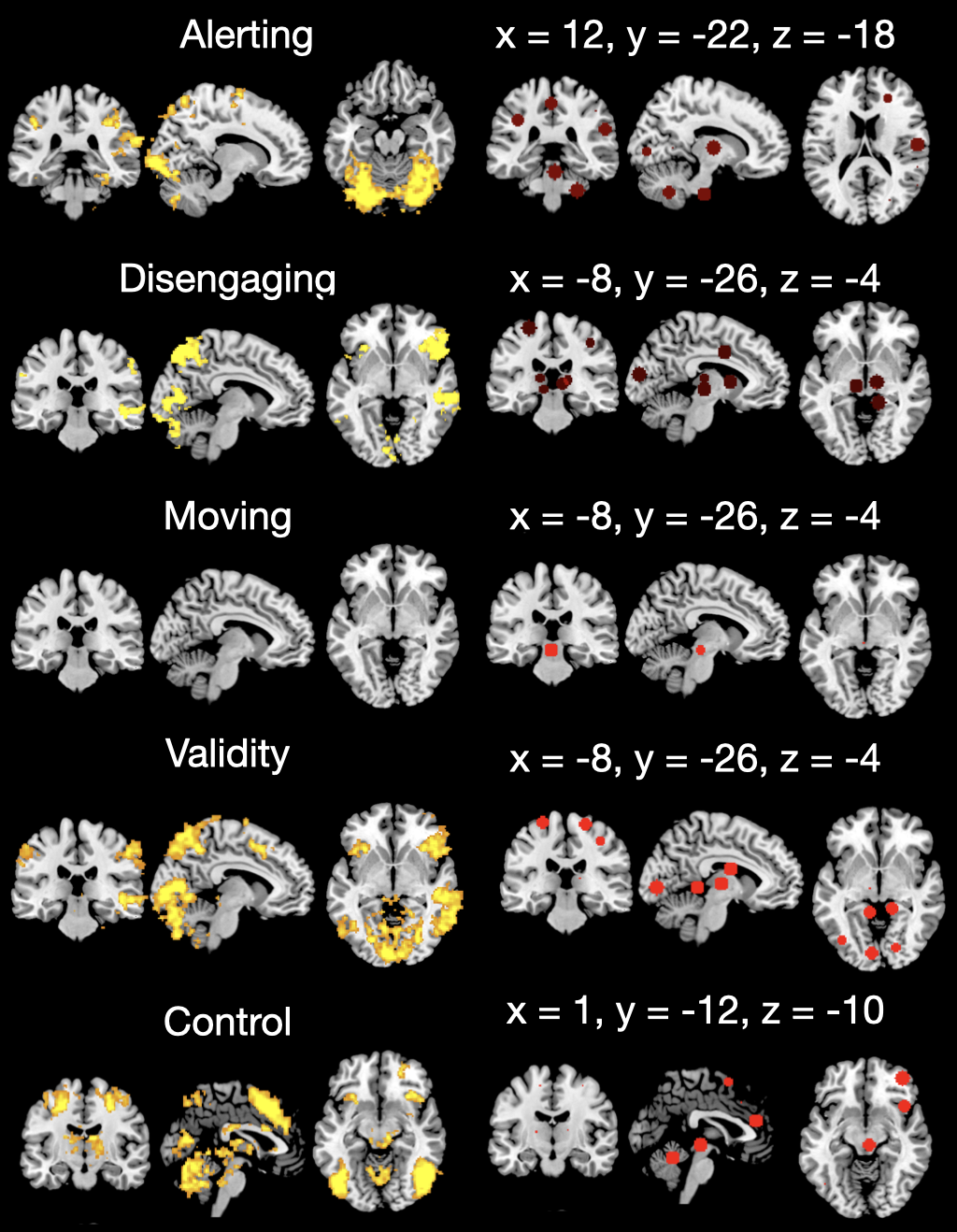
**

Figure S3: The column on the left shows volumetric task activations projected on a standard MNI template. The colum on the right shows the corresponding peak-location-ROI-map based on the results by Xuan et al. (2016). Settings for slice display are the same as in Xuan et al. (2016).

**T2. SPM results for volumetric assessment**

*Alerting*

We found 15 peak activations (>8mm apart) within the search volume for alerting, replicating 11/37 peak-activations from Xuan et al. (2016). The p-value is FWE-corrected at the voxel level within the search volume.

**
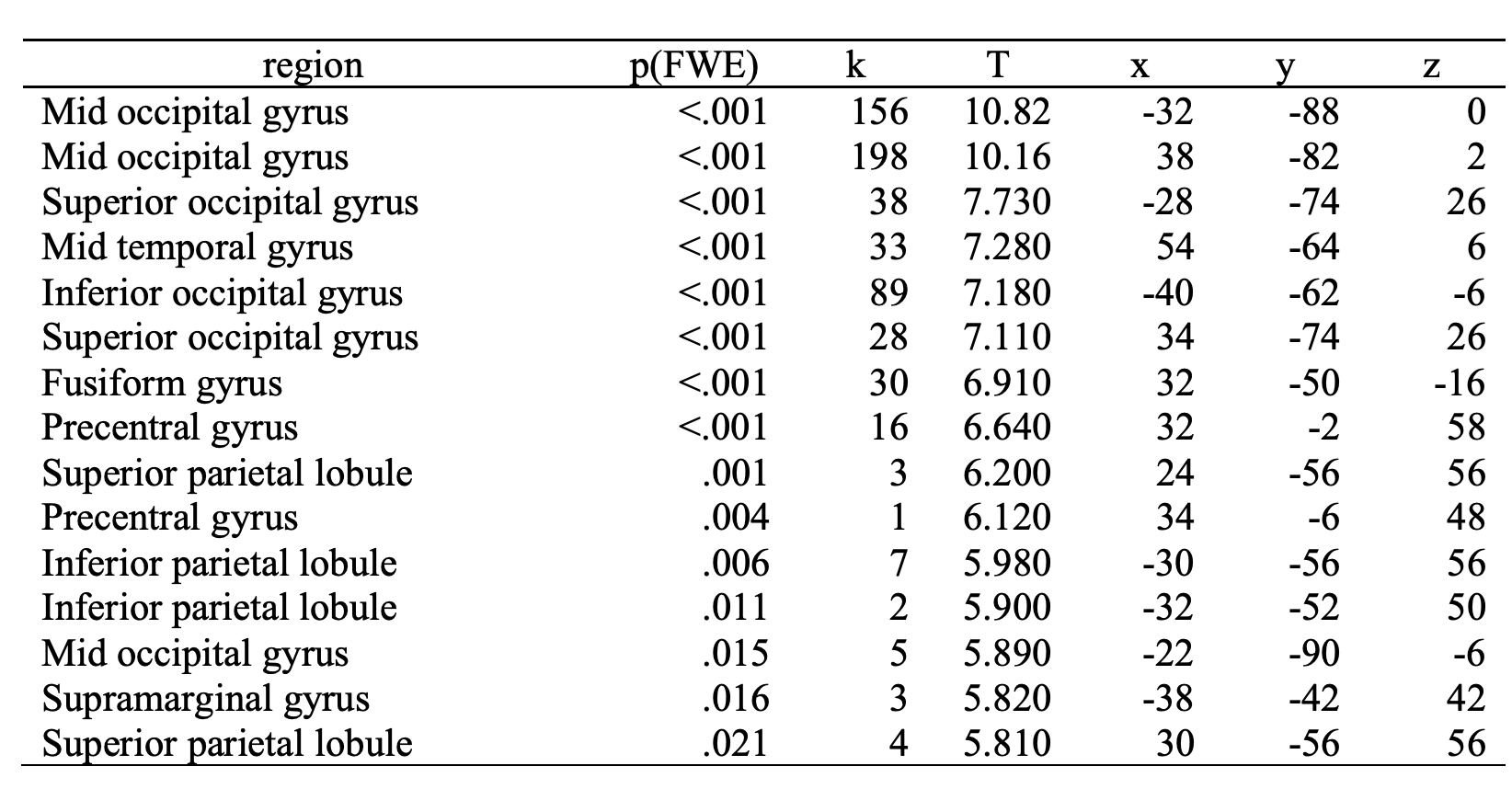
**

We did not confirm activation at coordinates with the following labels: Pons, Calcarine cortex, Precuneus, Postcentral gyrus, Cerebellum, Postcentral gyrus, Anterior insula, Rolandic operculum, Cerebellum, Anterior cingulate cortex, Anterior cingulate cortex, Anterior insula, Cerebellum, Cerebellum, Locus coeruleus, Mid frontal gyrus, Precentral gyrus, Precuneus, Putamen, Superior frontal gyrus, Supramarginal gyrus, Thalamus.

*Disengaging*

We found 1 peak activation (>8mm apart) within the search volume for Disengaging, replicating 1/16 peak-activations from Xuan et al. (2016). The p-value is FWE-corrected at the voxel level within the search volume.


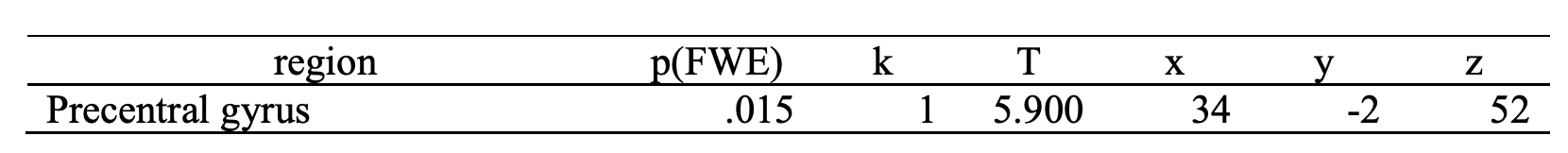


*Validity*

We found 11 peak activations (>8mm apart) within the search volume for Validity, replicating 8/20 peak-activations from Xuan et al. (2016). The p-value is FWE-corrected at the voxel level within the search volume.


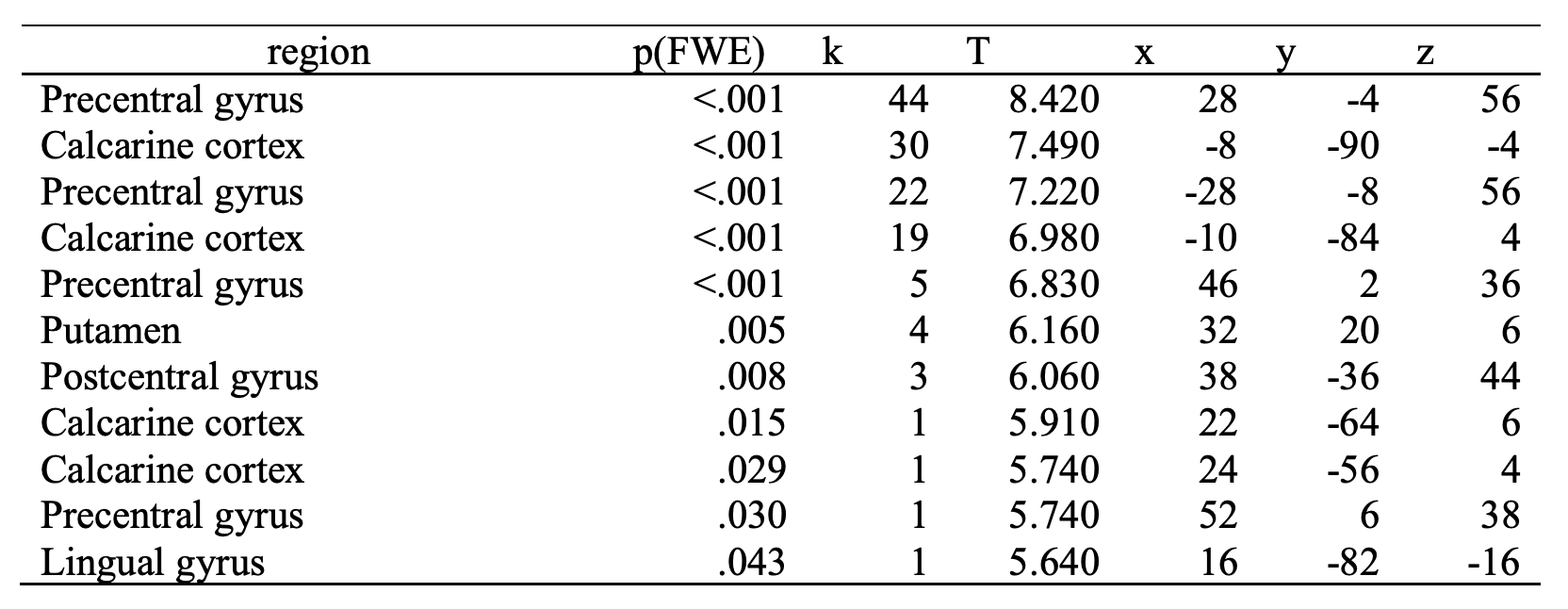


We did not confirm activation at coordinates with the following labels: Parahippocampal gyrus, Postcentral gyrus, Caudate nucleus, Thalamus, Precentral gyrus, Supplementary motor area, Cuneus, Inferior occipital gyrus, Fusiform gyrus, Thalamus

*Control*

We found 16 peak activations (>8mm apart) within the search volume for Control, replicating 15/25 peak-activations from Xuan et al. (2016). The p-value is FWE-corrected at the voxel level within the search volume.


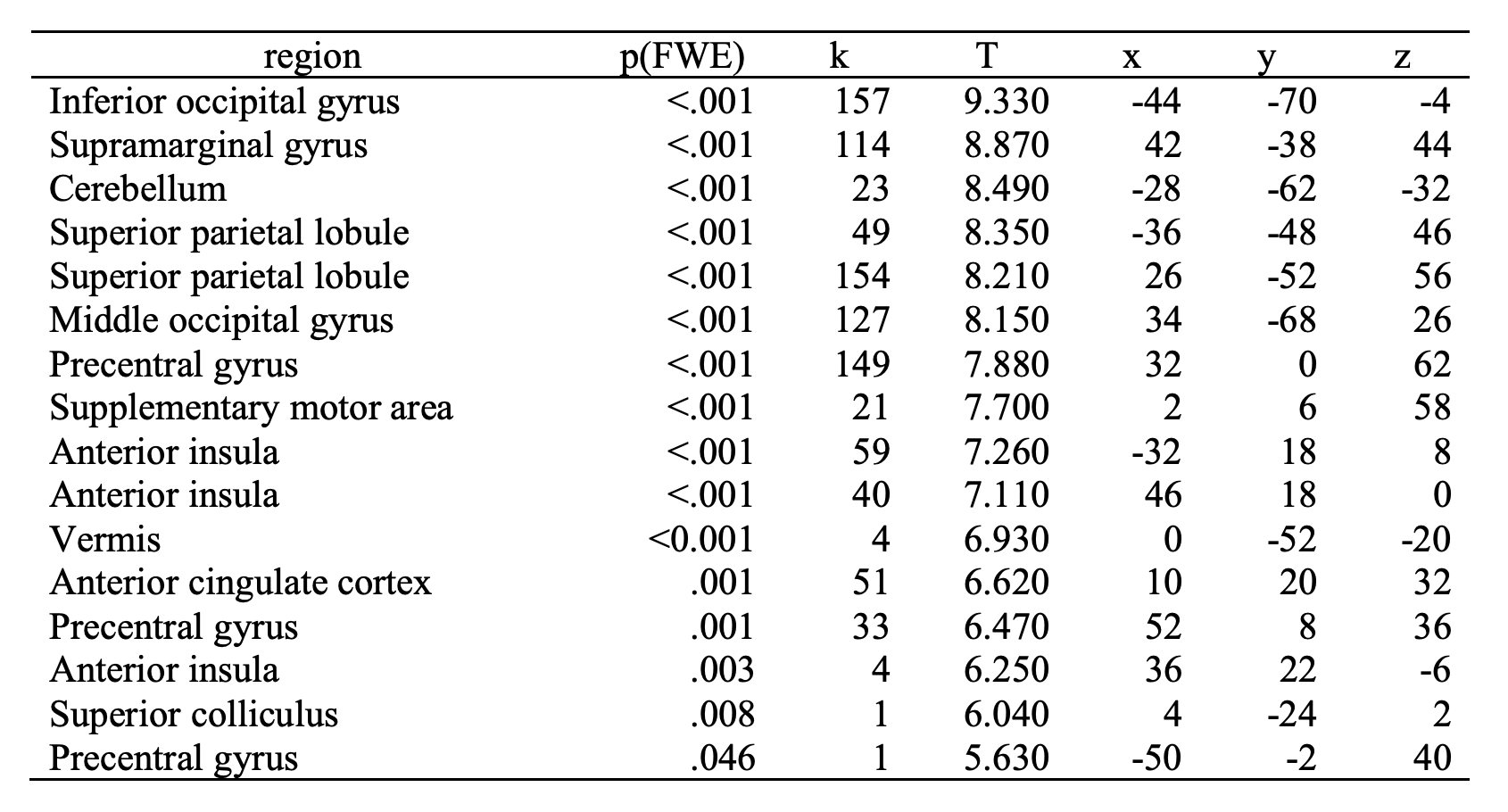


We did not confirm activation at coordinates with the following labels: Supramarginal gyrus, Precentral gyrus, Anterior cingulate cortex, Thalamus, Inferior frontal gyrus, Inferior parietal lobule, Cerebellum, Mid frontal gyrus, Superior frontal gyrus, Thalamus.

**S4-S7. Regression diagnostics**

We present diagnostics for the spatial regression models from the main text. We present a plot of fitted vs. actual values, a plot of the fitted values vs. the residuals, a histogram of the residuals, a plot of the residuals vs. lagged residuals (lag 1), and a probability plot for the residuals regarding normality. None of the diagnostics indicated problems for the regression analyses.

Figure S4: Regression diagnostics for Alerting

Figure S5: Regression diagnostics for Disengaging

Figure S6: Regression diagnostics for Moving and Engaging

Figure S7: Regression diagnostics for the Validity effect

Figure S8: Regression diagnostics for control

**S9-S10. Comparison with the Yeo networks**

We complement our main analysis with our own ICA-based ICN partition with a similar spatial correspondance analysis with the networks maps provided by Yeo et al. (2011).

Since the Yeo partitions are cortical only, the analysis was restricted to cortical grayordinates. Since the Yeo networks are binary (with each grayordinate belonging to exactly one network), a spatial regression analysis becomes difficult due to the rank deficiency of the design matrix. We therefore opted for bivariate comparisons between each Yeo network and each attention contrast by computing point biserial correlations (for one binary and one continuous variable).

Figure S9: Point biserial correlations between ANT activations and the seven Yeo networks. The darker shading highlights ICN with at least 5% shared variance with the respective ANT map. The order of ICN on the x-axis is in the same order as in Yeo et al. (2011).

Figure S10: Point biserial correlations between ANT activations and the seventeen Yeo networks. The darker shading highlights ICN with at least 5% shared variance with the respective ANT map. The order of ICN on the x-axis was reordered to present subnetworks belonging to the same higher order network next to each other.

**T2. Correlations between the ICN-contributions from the Yeo partitions to the ANT contrasts**

T2. The upper triangle gives correlations for the Yeo-7 partition, the lower triangle for the Yeo-17 partition. Please note: The correlations in the upper triangle are based on seven data points which restricts statistical power.

|  | Alerting | Disengaging | Moving | Validity | Control |
| --- | --- | --- | --- | --- | --- |
| Alerting |  | .347 | - .904* | .678 | .730 |
| Disengaging | .143 |  | .061 | .910* | .724 |
| Moving | - .908* | .159 |  | -.358* | -.442 |
| Validity | .578* | .877* | - .335 |  | .885* |
| Control | .558* | .717* | -. 304 | .839* |  |

**p<.05*
